# Clinical features of Cytomegalovirus retinitis in patients with acquired immunodeficiency syndrome and efficacy of the current therapy

**DOI:** 10.3389/fcimb.2023.1107237

**Published:** 2023-05-26

**Authors:** Qing Zhao, Ning-ning Li, You-xin Chen, Xin-yu Zhao

**Affiliations:** ^1^ Department of Ophthalmology, Peking Union Medical College Hospital, Chinese Academy of Medical Sciences, Beijing, China; ^2^ Key Laboratory of Ocular Fundus Diseases, Chinese Academy of Medical Sciences and Peking Union Medical College, Beijing, China; ^3^ Department of Operating Room, Peking Union Medical College Hospital, Chinese Academy of Medical Sciences, Beijing, China

**Keywords:** Cytomegalovirus retinitis, acquired immunodeficiency syndrome, clinical feature, efficacy, systematic review, meta-analysis

## Abstract

**Background:**

Cytomegalovirus retinitis (CMVR) is the most common and sight-threatening opportunistic retinal infection in patients with acquired immunodeficiency syndrome (AIDS) and several controversies remain to be settled. We aimed to summarize the current evidence and clarify the clinical features and prognosis of CMVR in AIDS patients.

**Methods:**

The databases PubMed, EMBASE, and Ovid from inception to April 2022 were searched to identify the relevant studies. R software version 3.6.3 was used to perform the statistical analyses. Results in proportion with 95% confidence interval (CI) were calculated using the Freeman-Tukey variant of arcsine square transformation.

**Results:**

We finally included 236 studies comprising 20,214 patients. CMVR in AIDS was male-dominated (88%, 95%CI 86%-89%), with 57% (95%CI 55%-60%) aged <41 years and 44% (95%CI 41%-47%) being bilaterally involved. CMVR was preponderant in AIDS patients with the following characteristics: white and non-Hispanic, homosexual, HIV RNA load ≥ 400 copies/mL, and CD4+ T-cells <50 cells/μL. The positivity of CMV-DNA in blood, aqueous humor, and vitreous humor was 66% (95%CI 52%-79%), 87% (95%CI 76%-96%), and 95% (95%CI 85%-100%), respectively. The most common symptoms were blurred vision (55%, 95%CI 46%-65%), followed by asymptomatic, visual field defect, and floaters. CMVR was first diagnosed and regarded as the clue to AIDS diagnosis in 9% (95%CI 6%-13%) of CMVR patients. Approximately 85% (95%CI 76%-93%) of the CMVR patients have received cART. CMVR remission was observed in 72%-92% of patients depending on the specific category of anti-CMV therapy. The general incidence of CMVR-related RD in the entire course was 24% (95%CI 18%-29%), of which most patients received PPV with SO or gas tamponade and the rate of anatomic success was 89% (95%CI 85%-93%).

**Conclusion:**

CMVR is a common opportunistic infection with diverse clinical features in AIDS patients, preponderant in those who are male, homosexual, or with CD4+ T-cells <50 cells/μL. Current therapies for CMVR and CMVR-related RD were shown to be effective. Early detection and routine ophthalmic screening should be promoted in AIDS patients.

**Systematic review registration:**

PROSPERO, identifier CRD42022363105.

## 1 Introduction

Cytomegalovirus retinitis (CMVR), which affects around 20%-40% of patients positive for human immunodeficiency virus (HIV) (Chen et al., 2017; Kim et al., 2017), is the most common end-stage opportunistic retinal infection in patients with acquired immunodeficiency syndrome (AIDS) (Griffiths et al., 2015). Combination antiretroviral therapy (cART) has reduced the risk of newly diagnosed CMVR by more than 90% in the developed world (Palella et al., 1998). However, the prevalence of CMVR in developing countries, such as South-East Asia, remains relatively high (Stewart, 2010). Furthermore, despite the widespread availability of cART, CMVR is still the leading cause of vision loss in AIDS patients and remains a serious and sight-threatening disease in countries with a high HIV burden (Thorne et al., 2006a). In a study of AIDS patients in five African and Asian countries, Heiden et al. (2007) found that 37% of CMVR eyes were blinded by the infection, suggesting a high rate of blindness in developing countries. Retinal detachment (RD) is perhaps the most devastating cause of visual acuity (VA) loss in CMVR patients (Thorne et al., 2006a).

CMVR is mainly characterized by brush-fire or hemorrhagic necrosis lesions, opaque granular lesions, and frosted branch angiitis (Port et al., 2018; Tadepalli et al., 2020). CMVR patients may only experience blurred vision or floaters as early symptoms (Singh et al., 2020), which are easily ignored. The diagnosis of CMVR is mainly based on a combination of medical history in susceptible individuals, clinical and laboratory findings, and imaging techniques (Port et al., 2017). Indirect fundoscopy with fully dilated pupils is the gold standard in CMVR diagnosis, and polymerase chain reaction (PCR) amplification of CMV-DNA from various specimens is also useful (Cunha Ade et al., 2002). Anti-CMV agents mainly include ganciclovir, valganciclovir, foscarnet, and cidofovir, administered either systemically (intravenous or oral route) or locally (intraocular ganciclovir implant or intravitreal injection). For CMVR-related RD, pars plana vitrectomy (PPV) with silicone oil (SO) tamponade has been the most preferred treatment (Wong et al., 2014).

However, numerous controversies still need to be addressed. Firstly, identifying CMVR as the clue to AIDS diagnosis may help to initiate cART as early as possible (Chu and Selwyn, 2010), but very few studies have focused on this issue. Secondly, the previously reported incidence of CMVR symptoms in AIDS patients varied remarkably. For example, the incidence of blurred vision, floaters, and scotoma varied from 13% to 100% (Magone et al., 1997; Gharai et al., 2008), 2% to 65% (Gharai et al., 2008; Singh et al., 2020), and 4% to 51% (Dhillon et al., 1993; Singh et al., 2020), respectively. Furthermore, the incidence of other symptoms, especially being asymptomatic, remains largely inconclusive. Thirdly, multiple studies have evaluated the efficacy of various anti-CMV agents (Moyle et al., 1992; Diaz-Llopis et al., 1994; Roth et al., 1999), but the results remain inconsistent. The CMVR remission rate ranged from 9% to 77% for intravenous ganciclovir and 3% to 82% for intravitreal ganciclovir. These controversies severely restrict the clinical management and academic experience sharing of CMVR in AIDS patients. Fourthly, CMVR-related RD is of increasing concern to ophthalmologists, but its incidence varied from 23% to 45% (Kempen et al., 2001; Huang et al., 2015) and the anatomic success rate of PPV varied largely from 57% to 100% (Azen et al., 1998; Martidis et al., 2002; Scott et al., 2003; Almeida et al., 2015). Fifthly, the limited sample size of previous studies may limit their conclusions being a solid reference. Finally, several authors have suggested that a proper meta-analysis be performed to summarize all the current evidence regarding CMVR in AIDS patients.

However, no such meta-analysis has been conducted yet, and a comprehensive analysis is urgently needed. Our aim in this meta-analysis is to summarize information and help addressing existing controversies on the demographics, clinical features, treatment, and prognosis of CMVR. The results may be used to inform clinical practice by providing ophthalmologists with a comprehensive overview of the current state of knowledge on this condition.

## 2 Methods

This study was conducted in accordance with the Preferred Reporting Items for Systematic Reviews and Meta-analyses (PRISMA) statement (Page et al., 2021) and was pre-registered on PROSPERO (identifier CRD42022363105).

### 2.1 Search strategy and study identification

PubMed, EMBASE, and Ovid were searched to identify relevant studies published from inception to April 2022. The language was restricted to English with no restriction on publication date. The following keywords and MeSH terms were searched: cytomegalovirus retinitis, human immunodeficiency virus, acquired immunodeficiency syndrome, and multiple synonyms. A detailed search strategy for the PubMed database was developed and then adapted for searching studies in other databases (see **Supplementary table 1**). References of all included studies were manually searched to identify additional related studies.

### 2.2 Inclusion criteria and exclusion criteria

The eligibility criteria were as follows: 1) research data on CMVR in AIDS patients; and 2) full-text available. Exclusion criteria included: 1) studies with a sample size <5; and 2) literature reviews, systematic reviews, meta-analysis, editorial, conference abstract, or technical notes; and 3) studies on animal or cadaver subjects.

### 2.3 Data extraction and quality assessment

After removing duplicated publications, two researchers (QZ and XYZ) independently screened the titles and abstracts of the retrievals to identify eligible studies. Full texts of all potentially eligible studies were then read for further discrimination. The following data were extracted: *1) Study characteristics:* the first author, publication year, study design, group size (patients and eyes), and duration of follow-up. *2) Patient characteristics:* age, gender, ethnicity, HIV transmission route, HIV RNA load, CMV load, CMV positivity, extraocular CMV infection, and other systemic co-morbidities. *3) CMVR characteristics:* laterality, CMVR as a clue to AIDS diagnosis, symptoms, signs, retina area involved, location, baseline and final VA, therapy, prognosis, and ocular complications. “Retina area involved” was categorized into “0–25%”, “25–50%”, and “50%-100%”. “Location” was grouped as “central type” or “peripheral type”. The “central type” was defined as CMVR involving the region of one optic disc diameter (1500 μm) from the edge of the optic disc or two optic disc diameters (3000 μm) from the fovea; the “peripheral type” was defined as CMVR involving regions other than those categorized in the central type (Kim et al., 2017). The baseline VA was categorized into “better than 20/50”, “20/50 to 20/160”, and “20/200 or worse”. If encounter missing data, we will contact the corresponding author of the original study for further data. Any discrepancies were resolved by discussion or consulting the third researcher (YXC).

For quality assessment, we used the Newcastle-Ottawa Scale to assess the non-randomized studies. Studies with a score of 6 or higher were considered moderate to high quality. The randomized studies were assessed using the criteria outlined in Chapter 8 of the *Cochrane Handbook for Systematic Reviews of Interventions*. Two researchers (QZ and XYZ) independently scored each study, and any disagreements were resolved through discussion or by consulting a third researcher (YXC).

### 2.4 Statistical methods

We employed R version 3.6.3 (R Foundation for Statistical Computing, Vienna, Austria) for statistical analyses. The Freeman-Tukey variant of arcsine square transformation was applied to calculate proportions with 95% confidence interval (CI) and to build forest plots. The heterogeneity across studies was evaluated using the chi-square test and I^2^ statistics. If heterogeneity was low (P>0.1, I^2^<50%), the fixed-effect model would be chosen. When heterogeneity was significant (P<0.1, I^2^>50%), sensitivity analysis and subgroup analysis would be performed to identify its source, and a random-effect model would be applied when encountering clinical homogeneity. We will perform descriptive analysis if clinical heterogeneity was found in the result of the meta-analysis. The publication bias was evaluated using the funnel plot of the Egger test. P<0.05 was considered statistically significant.

## 3 Results

### 3.1 Study characteristics

**Figure 1:**
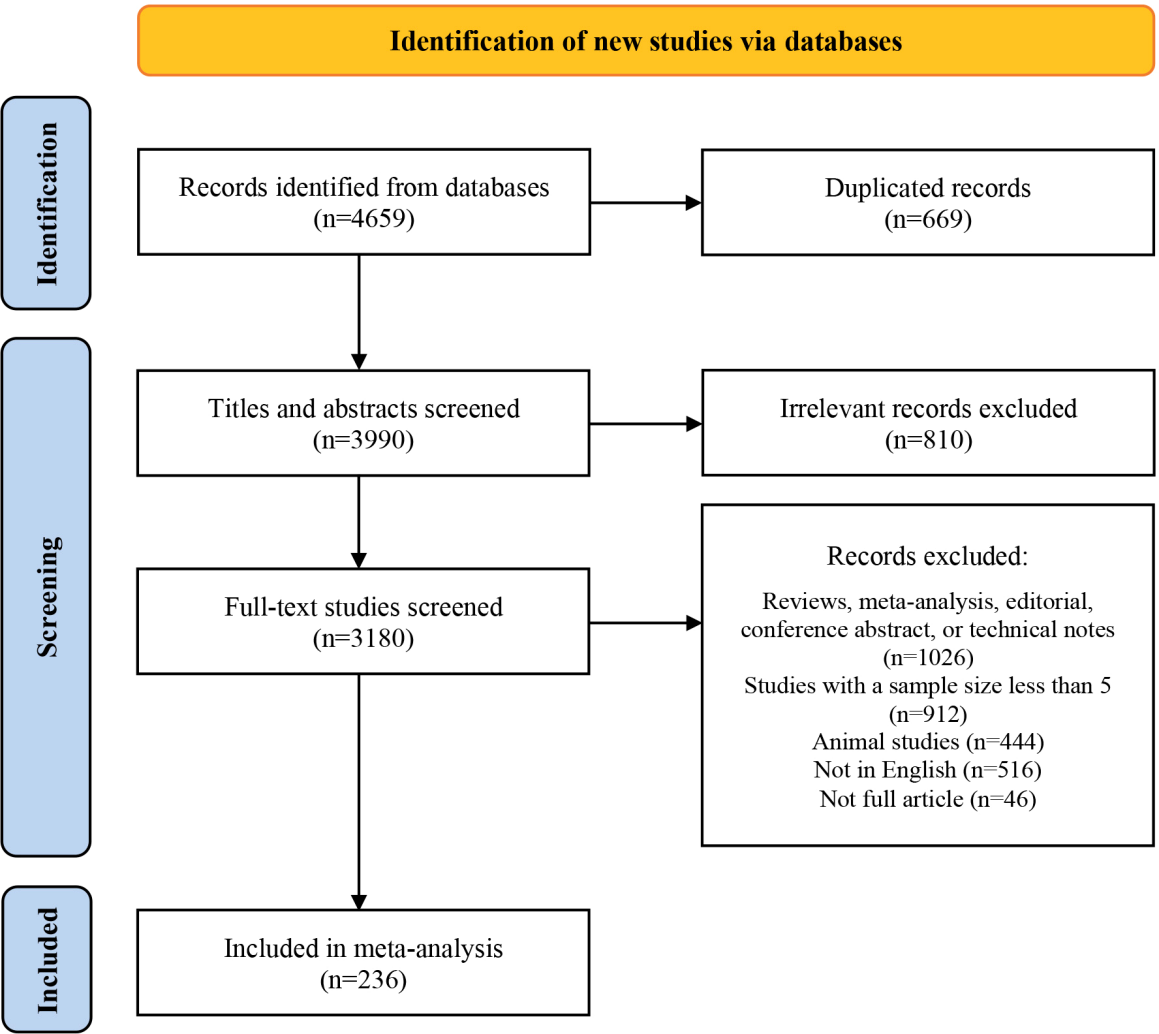
PRISMA Flowchart of Study Selection.

Initially, we identified 4,659 studies. After removing duplicates and screening titles and abstracts, we ultimately reviewed the full texts of 3,180 articles. Of these, 236 studies comprising 20,214 patients were eligible for this study. The screening process was presented using the PRISMA flow chart (see **Figure 1**), and the main characteristics of all included studies were shown in **Supplementary table 2**.

### 3.2 Demographics

CMVR was male-dominated (88%, 95%CI 86%-89%, I^2 =^ 86%), with 57% (95%CI 55%-60%, I^2 =^ 31%) aged <41 years and 44% (95%CI 41%-47%, I^2 =^ 86%) bilaterally involved. CMVR was more prevalent in AIDS patients with the following characteristics: white and non-Hispanic (60%, 95%CI 55%-64%, I^2 =^ 95%), homosexual (70%, 95%CI 67%-74%, I^2 =^ 93%), HIV RNA load ≥ 400 copies/mL (82%, 95%CI 70%-91%, I^2 =^ 96%), or CD4^+^ T-cells <50 cells/μL (78%, 95%CI 71%-85%, I^2 =^ 86%).

**Figure 2:**
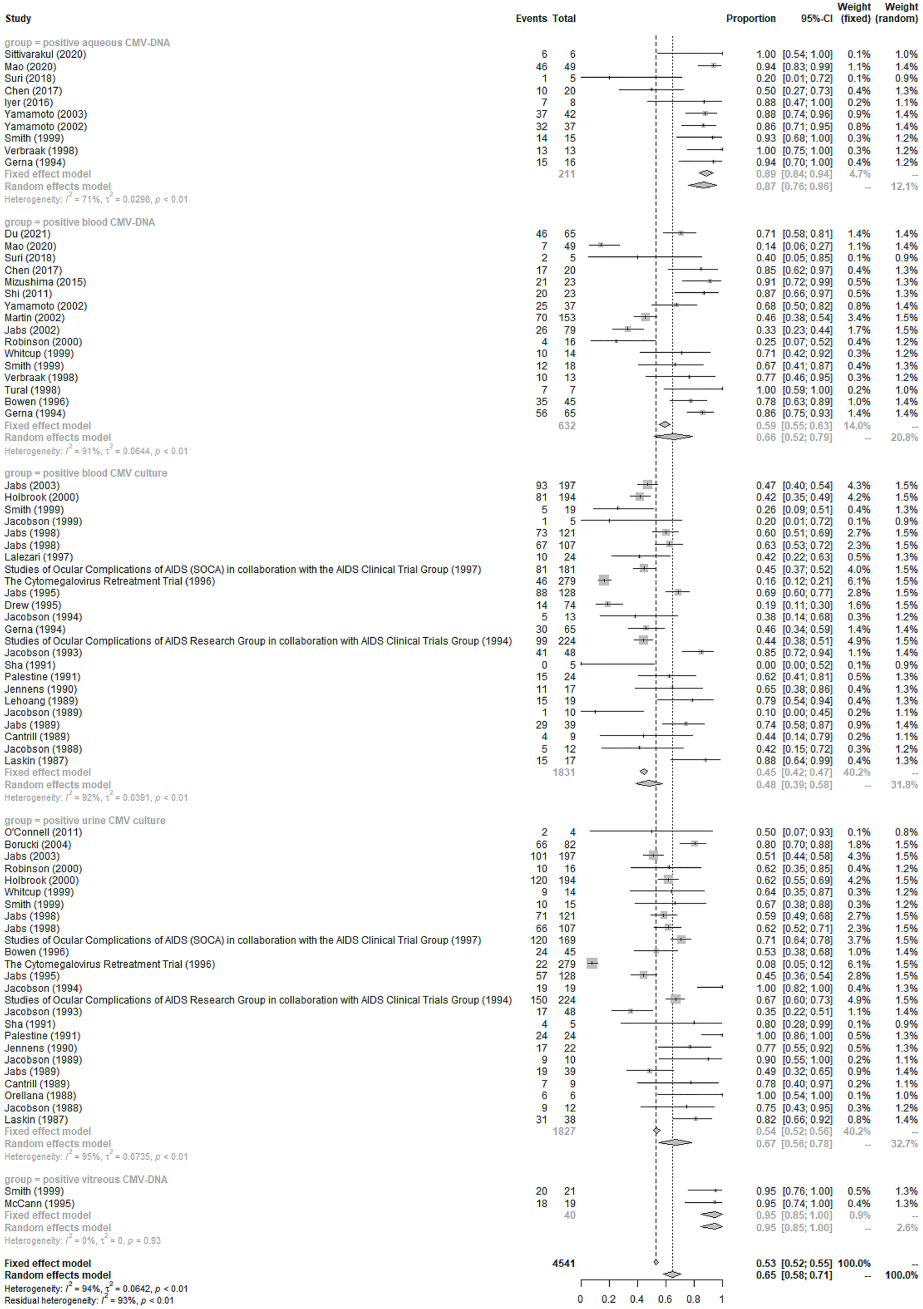
Forest Plots of the Positivity of CMV-DNA and CMV culture of CMVR in AIDS Patients.

Positive urine or blood CMV culture was found in 67% (95%CI 56%-78%, I^2 =^ 95%) and 48% (95%CI 39%-58%, I^2 =^ 92%) of the included CMVR patients, respectively (see **Figure 2**). Blood, aqueous humor, and vitreous humor CMV-DNA positivity was 66% (95%CI 52%-79%, I^2 =^ 91%), 87% (95%CI 76%-96%, I^2 =^ 71%), and 95% (95%CI 85%-100%, I^2 =^ 0%), respectively (see **Figure 2**). The pooled incidence of extraocular CMV infection in 46 studies (3,635 patients) was 18% (95%CI 15%-22%, I^2 =^ 85%). The pooled incidence of other systemic co-morbidities in CMVR patients was summarized in **Table 1**.

**Table 1:** The pooling results of demographic characteristics of CMVR in aIDS patients.

Category	No. of studies	Pooled incidence	95% CI		*P*-value	*I* ^2^	Sensitivity analysis	Selected model
Age								
*< 41*	*29*	*57%*	*55%*	*60%*	*P=0.06*	*31%*	*Negative*	*Fixed-effect model*
*≥ 41*	*29*	*43%*	*40%*	*46%*	*P=0.01*	*41%*	*Negative*	*Fixed-effect model*
Gender								
*Male*	*173*	*88%*	*86%*	*89%*	*P<0.01*	*86%*	*Negative*	*Random-effect model*
*Female*	*173*	*12%*	*11%*	*14%*	*P<0.01*	*86%*	*Negative*	*Random-effect model*
Ethnicity								
*White, non-Hispanic*	*60*	*60%*	*55%*	*64%*	*P<0.01*	*95%*	*Negative*	*Random-effect model*
*Black, non-Hispanic*	*42*	*25%*	*20%*	*30%*	*P<0.01*	*95%*	*Negative*	*Random-effect model*
*Hispanic*	*33*	*16%*	*12%*	*19%*	*P<0.01*	*91%*	*Negative*	*Random-effect model*
*Asian*	*19*	*27%*	*10%*	*48%*	*P<0.01*	*98%*	*Negative*	*Random-effect model*
Laterality of involvement								
*Bilateral*	*126*	*44%*	*41%*	*47%*	*P<0.01*	*86%*	*Negative*	*Random-effect model*
*Unilateral*	*126*	*56%*	*53%*	*59%*	*P<0.01*	*86%*	*Negative*	*Random-effect model*
HIV RNA load								
*< 400 copies/mL*	*17*	*18%*	*12%*	*26%*	*P<0.01*	*91%*	*Negative*	*Random-effect model*
*≥ 400 copies/mL*	*17*	*82%*	*70%*	*91%*	*P<0.01*	*96%*	*Negative*	*Random-effect model*
Route of HIV transmission								
*Homosexual*	*67*	*70%*	*67%*	*74%*	*P<0.01*	*93%*	*Negative*	*Random-effect model*
*Intravenous drug use*	*59*	*12%*	*10%*	*15%*	*P<0.01*	*93%*	*Negative*	*Random-effect model*
*Heterosexual*	*38*	*18%*	*14%*	*22%*	*P<0.01*	*93%*	*Negative*	*Random-effect model*
*Homosexual & intravenous drug use*	*12*	*2%*	*2%*	*3%*	*P=0.03*	*49%*	*Negative*	*Fixed-effect model*
*Contaminated blood transfusion*	*12*	*3%*	*1%*	*5%*	*P<0.01*	*71%*	*Negative*	*Random-effect model*
CD4+ T-cells at CMVR diagnosis								
*< 50 cells/μL*	*22*	*78%*	*71%*	*85%*	*P<0.01*	*86%*	*Negative*	*Random-effect model*
*≥ 50 cells/μL*	*22*	*22%*	*13%*	*31%*	*P<0.01*	*91%*	*Negative*	*Random-effect model*
Blood CMV load								
*< 400 copies/mL*	*8*	*62%*	*43%*	*80%*	*P<0.01*	*98%*	*Negative*	*Random-effect model*
*≥ 400 copies/mL*	*8*	*38%*	*20%*	*57%*	*P<0.01*	*98%*	*Negative*	*Random-effect model*
CMV positivity								
*Positive urine CMV culture*	*25*	*67%*	*56%*	*78%*	*P<0.01*	*95%*	*Negative*	*Random-effect model*
*Positive blood CMV culture*	*24*	*48%*	*39%*	*58%*	*P<0.01*	*92%*	*Negative*	*Random-effect model*
*Positive blood CMV-DNA*	*16*	*66%*	*52%*	*79%*	*P<0.01*	*91%*	*Negative*	*Random-effect model*
*Positive aqueous CMV-DNA*	*10*	*87%*	*76%*	*96%*	*P<0.01*	*71%*	*Negative*	*Random-effect model*
*Positive vitreous CMV-DNA*	*2*	*95%*	*85%*	*100%*	*P=0.93*	*0%*	*Negative*	*Random-effect model*
Extraocular CMV infection	46	18%	15%	22%	*P*<0.01	85%	Negative	Random-effect model
Other systemic co-morbidities								
*Pneumocystis carinii pneumonia*	*20*	*38%*	*28%*	*50%*	*P<0.01*	*84%*	*Negative*	*Random-effect model*
*Tuberculosis*	*19*	*19%*	*12%*	*27%*	*P<0.01*	*81%*	*Negative*	*Random-effect model*
*Kaposi sarcoma*	*16*	*25%*	*15%*	*35%*	*P<0.01*	*78%*	*Negative*	*Random-effect model*
*Cryptosporidiosis*	*10*	*6%*	*4%*	*10%*	*P=0.07*	*43%*	*Negative*	*Fixed-effect model*
*Candidiasis*	*8*	*25%*	*10%*	*43%*	*P<0.01*	*90%*	*Negative*	*Random-effect model*
*Lymphoma*	*7*	*8%*	*2%*	*16%*	*P=0.06*	*50%*	*Negative*	*Random-effect model*
*Central nervous system toxoplasmosis*	*6*	*5%*	*2%*	*11%*	*P=0.05*	*55%*	*Negative*	*Random-effect model*
*Herpes simplex virus infection*	*5*	*17%*	*5%*	*33%*	*P<0.01*	*85%*	*Negative*	*Random-effect model*
*Herpes zoster virus infection*	*4*	*3%*	*1%*	*7%*	*P=0.19*	*38%*	*Negative*	*Fixed-effect model*
*Syphilis*	*4*	*26%*	*12%*	*42%*	*P<0.01*	*78%*	*Negative*	*Random-effect model*
*Diabetes mellitus*	*3*	*5%*	*1%*	*11%*	*P=0.64*	*0%*	*Negative*	*Fixed-effect model*
*Hepatitis*	*3*	*31%*	*8%*	*60%*	*P<0.01*	*87%*	*Negative*	*Random-effect model*
*HIV-wasting syndrome*	*2*	*11%*	*3%*	*21%*	*P=0.18*	*45%*	*Negative*	*Fixed-effect model*

AIDS, acquired immunodeficiency syndrome; CI, confidence interval; CMVR, cytomegalovirus retinitis; HIV, human immunodeficiency virus.

### 3.3 Clinical features

**Figure 3:**
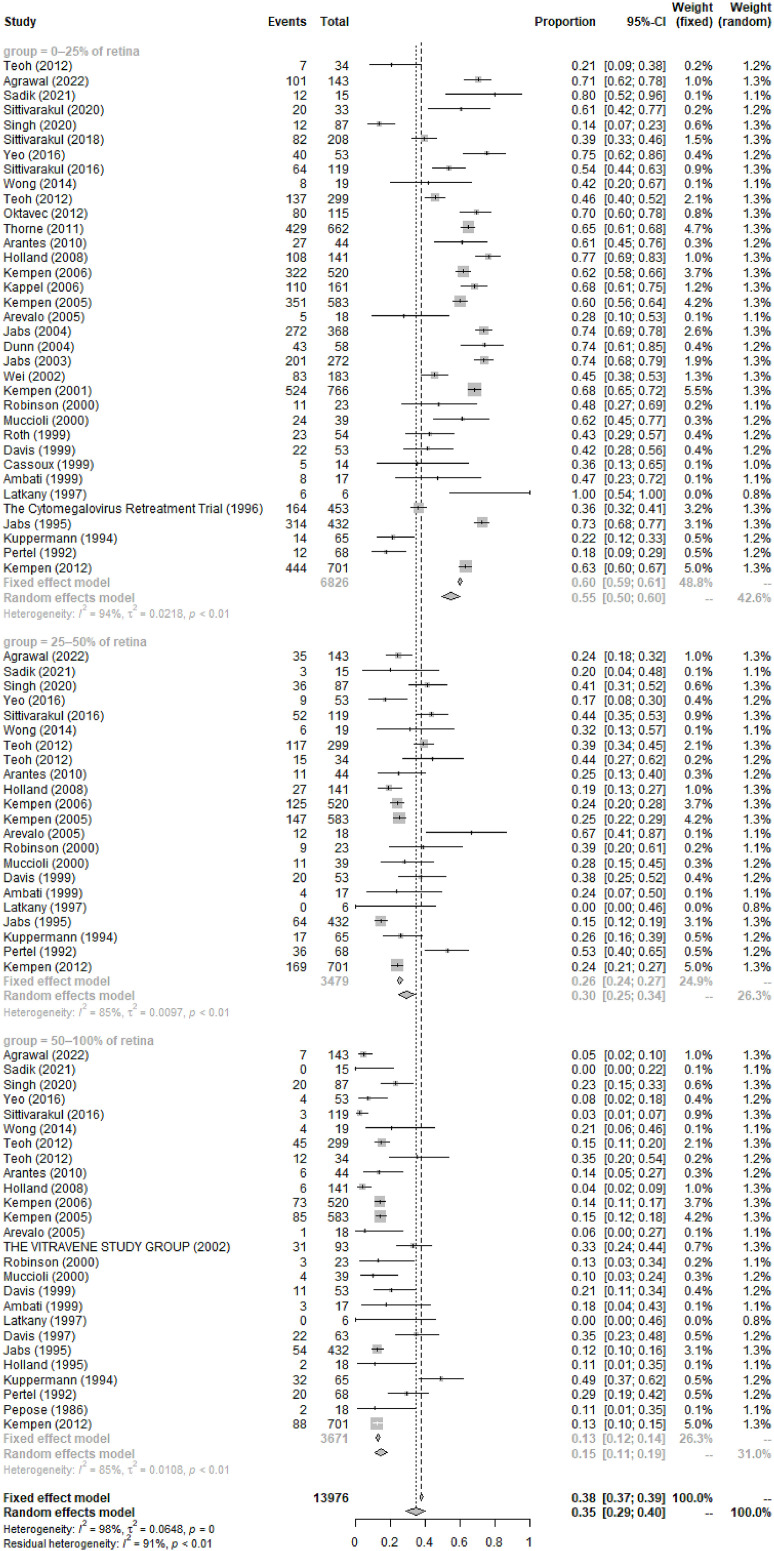
Forest Plots of the Retinal Area Involved of CMVR in AIDS Patients.

The most common symptoms of CMVR were blurred vision (55%, 95%CI 46%-65%, I^2 =^ 89%), followed by asymptomatic, visual field defect, floaters, photophobia, watering, and pain. The pooled incidence of these symptoms was summarized in **Table 2**. In 6 studies comprising 258 patients, the pooled incidence of CMVR as the clue to AIDS diagnosis in CMVR patients was 9% (95%CI 6%-13%, I^2 =^ 0%). The pooled results of the retina area involved, retinitis location, and baseline VA were also shown in **Table 2**; **Figure 3**.

**Table 2:** The Pooling results of the incidence of clinical features of CMVR in aIDS patients.

Category	No. of studies	Pooled incidence	95% CI		*P*-value	*I* ^2^	Sensitivity analysis	Selected model
Visual symptoms								
*Blurred vision*	*22*	*55%*	*46%*	*65%*	*P<0.01*	*89%*	*Negative*	*Random-effect model*
*Floaters*	*17*	*20%*	*12%*	*29%*	*P<0.01*	*91%*	*Negative*	*Random-effect model*
*Asymptomatic*	*14*	*29%*	*21%*	*37%*	*P<0.01*	*84%*	*Negative*	*Random-effect model*
*Visual field defect*	*9*	*21%*	*10%*	*35%*	*P<0.01*	*91%*	*Negative*	*Random-effect model*
*Photophobia*	*5*	*20%*	*8%*	*34%*	*P=0.03*	*64%*	*Negative*	*Random-effect model*
*Pain*	*4*	*2%*	*0%*	*5%*	*P=0.05*	*63%*	*Negative*	*Random-effect model*
*Watering*	*2*	*13%*	*1%*	*33%*	*P=0.61*	*0%*	*Negative*	*Fixed-effect model*
CMVR as the index diagnosis for AIDS	6	9%	6%	13%	*P*=0.58	0%	Negative	Fixed-effect model
Signs								
*Vitreous cells*	*15*	*33%*	*21%*	*47%*	*P<0.01*	*96%*	*Negative*	*Random-effect model*
*Anterior chamber cells*	*13*	*25%*	*12%*	*39%*	*P<0.01*	*97%*	*Negative*	*Random-effect model*
*Optic disk edema*	*7*	*26%*	*8%*	*49%*	*P<0.01*	*93%*	*Negative*	*Random-effect model*
*Frosted branch angiitis*	*7*	*9%*	*5%*	*14%*	*P=0.06*	*50%*	*Negative*	*Random-effect model*
*Macular edema*	*6*	*38%*	*9%*	*71%*	*P<0.01*	*97%*	*Negative*	*Random-effect model*
*Fulminant retinitis*	*4*	*28%*	*22%*	*35%*	*P=0.63*	*0%*	*Negative*	*Fixed-effect model*
*Hemorrhagic retinal lesions*	*4*	*31%*	*16%*	*48%*	*P<0.01*	*92%*	*Negative*	*Random-effect model*
*Keratic precipitates*	*4*	*11%*	*1%*	*26%*	*P<0.01*	*90%*	*Negative*	*Random-effect model*
*Retinal arteritis*	*4*	*26%*	*9%*	*47%*	*P<0.01*	*80%*	*Negative*	*Random-effect model*
*Epiretinal membrane*	*4*	*10%*	*1%*	*26%*	*P<0.01*	*97%*	*Negative*	*Random-effect model*
*Granular retinitis*	*3*	*35%*	*25%*	*46%*	*P=0.12*	*52%*	*Negative*	*Random-effect model*
*Cotton-wool spots*	*3*	*34%*	*22%*	*48%*	*P<0.01*	*88%*	*Negative*	*Random-effect model*
*Widespread vascular occlusion*	*3*	*14%*	*0%*	*73%*	*P<0.01*	*94%*	*Negative*	*Random-effect model*
*Posterior synechiae*	*2*	*6%*	*4%*	*8%*	*P=0.26*	*20%*	*Negative*	*Fixed-effect model*
Retina area involved								
*0–25%*	*35*	*55%*	*50%*	*60%*	*P<0.01*	*94%*	*Negative*	*Random-effect model*
*25–50%*	*22*	*30%*	*25%*	*34%*	*P<0.01*	*85%*	*Negative*	*Random-effect model*
*50–100%*	*26*	*15%*	*11%*	*19%*	*P<0.01*	*85%*	*Negative*	*Random-effect model*
Location								
*Central type*	*74*	*49%*	*45%*	*53%*	*P<0.01*	*92%*	*Negative*	*Random-effect model*
*Peripheral type*	*74*	*51%*	*47%*	*55%*	*P<0.01*	*92%*	*Negative*	*Random-effect model*
Baseline visual acuity								
*Better than 20/50*	*20*	*64%*	*51%*	*75%*	*P<0.01*	*93%*	*Negative*	*Random-effect model*
*20/50 to 20/160*	*27*	*18%*	*14%*	*24%*	*P<0.01*	*91%*	*Negative*	*Random-effect model*
*20/200 or worse*	*17*	*18%*	*13%*	*23%*	*P<0.01*	*70%*	*Negative*	*Random-effect model*

AIDS, acquired immunodeficiency syndrome; CI, confidence interval; CMVR, cytomegalovirus retinitis.

### 3.4 Treatment and prognosis

A total of 85% (95%CI 76%-93%, I^2 =^ 98%) of 8,724 CMVR patients in 72 studies have ever received cART either at CMVR diagnosis or at enrollment. The pooled incidence of systemic, local or combinational anti-CMV therapy was 45% (95%CI 42%-48%, I^2=^ 98%), 35% (95%CI 29%-40%, I^2 =^ 99%), and 20% (95%CI 11%-29%, I^2 =^ 99%), respectively. Detailed therapy in the induction and maintenance stage was shown in **Supplementary Table 3**. The prognosis after receiving certain anti-CMV agents was summarized in **Table 3**. The CMVR recurrence rate was 16% (95%CI 4%-31%, I^2 =^ 78%) after achieving immune recovery and discontinuing maintenance anti-CMV therapy. The all-cause mortality rate of CMVR patients was 36% (95%CI 28%-44%, I^2 =^ 96%). Immune recovery uveitis (IRU) was observed in 26% (95%CI 19%-34%, I^2 =^ 95%) of 3748 CMVR patients in 28 studies after cART initiation and immune recovery. The specific postoperative complications after intraocular ganciclovir implants were summarized in **Supplementary Table 4**.

**Table 3:** The pooling results of the therapy and prognosis of CMVR in aIDS patients.

Category	No. of studies	Pooled incidence	95% CI		*P*-value	*I* ^2^	Sensitivity analysis	Selected model
Anti-HIV therapy during the entire courses								
*cART*	*72*	*85%*	*76%*	*93%*	*P<0.01*	*98%*	*Negative*	*Random-effect model*
*ART, no cART*	*31*	*6%*	*1%*	*14%*	*P<0.01*	*95%*	*Negative*	*Random-effect model*
*No ART*	*51*	*9%*	*4%*	*15%*	*P<0.01*	*95%*	*Negative*	*Random-effect model*
Anti-CMV therapy								
*Systemic*	*118*	*45%*	*42%*	*48%*	*P<0.01*	*98%*	*Negative*	*Random-effect model*
*Local*	*69*	*35%*	*29%*	*40%*	*P<0.01*	*99%*	*Negative*	*Random-effect model*
*Combinational*	*20*	*20%*	*11%*	*29%*	*P<0.01*	*99%*	*Negative*	*Random-effect model*
Prognosis								
CMVR remission								
*Intraocular ganciclovir implant*	*2*	*89%*	*42%*	*100%*	*P<0.01*	*96%*	*Negative*	*Random-effect model*
*Intravitreal ganciclovir*	*4*	*83%*	*57%*	*99%*	*P<0.01*	*78%*	*Negative*	*Random-effect model*
*Intravenous ganciclovir*	*11*	*90%*	*79%*	*98%*	*P<0.01*	*88%*	*Negative*	*Random-effect model*
*Intravenous and intravitreal ganciclovir*	*3*	*72%*	*39%*	*97%*	*P=0.04*	*68%*	*Negative*	*Random-effect model*
*Intravitreal foscarnet*	*3*	*72%*	*28%*	*100%*	*P<0.01*	*88%*	*Negative*	*Random-effect model*
*Intravenous foscarnet*	*3*	*92%*	*83%*	*98%*	*P=0.75*	*0%*	*Negative*	*Fixed-effect model*
CMVR progression								
*Intraocular ganciclovir implant*	*6*	*17%*	*11%*	*24%*	*P=0.02*	*64%*	*Negative*	*Random-effect model*
*Intravitreal ganciclovir*	*5*	*37%*	*3%*	*81%*	*P<0.01*	*97%*	*Negative*	*Random-effect model*
*Intravenous ganciclovir*	*15*	*37%*	*24%*	*52%*	*P<0.01*	*92%*	*Negative*	*Random-effect model*
*Oral ganciclovir*	*4*	*56%*	*23%*	*86%*	*P<0.01*	*98%*	*Negative*	*Random-effect model*
*Intravenous foscarnet*	*3*	*7%*	*2%*	*13%*	*P=0.87*	*0%*	*Negative*	*Fixed-effect model*
*Intravitreal cidofovir*	*2*	*9%*	*4%*	*16%*	*P=0.21*	*36%*	*Negative*	*Fixed-effect model*
*Intravenous cidofovir*	*2*	*52%*	*41%*	*63%*	*P=0.16*	*48%*	*Negative*	*Fixed-effect model*
CMVR recurrence after discontinuing maintenance anti-CMV therapy	*7*	*16%*	*4%*	*31%*	*P<0.01*	*78%*	*Negative*	*Random-effect model*
All-cause mortality	46	36%	28%	44%	*P*<0.01	96%	Negative	Random-effect model
Incidence of IRU^a^	28	26%	19%	34%	*P*<0.01	95%	Negative	Random-effect model

a IRU represents an increase in or new-onset of intraocular inflammation in the anterior chamber or vitreous of an eye with CMVR coincident with immune recovery after initiating cART.

AIDS, acquired immunodeficiency syndrome; ART, antiretroviral therapy; cART, combination antiretroviral therapy; CI, confidence interval; CMV, cytomegalovirus; CMVR, cytomegalovirus retinitis; IRU, immune recovery uveitis.

### 3.5 CMVR-related RD

**Figure 4:**
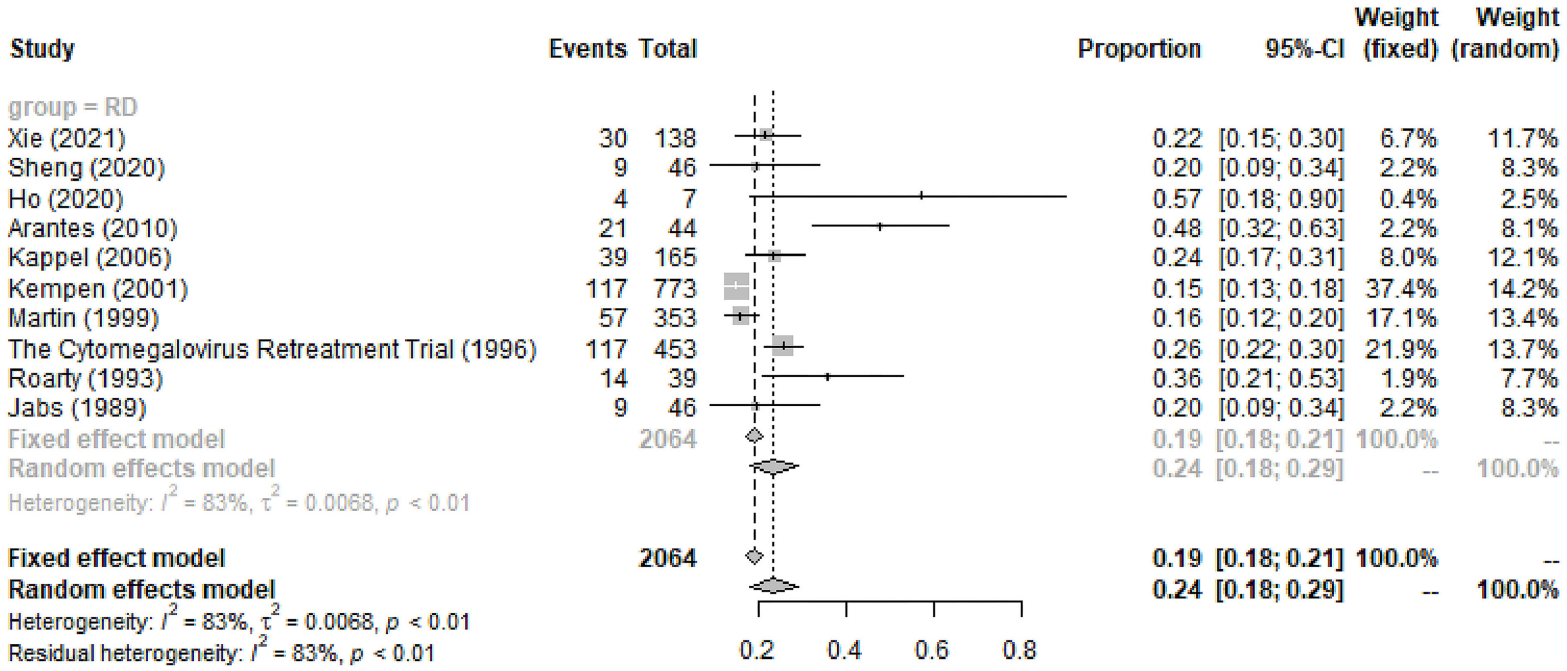
Forest Plots of the General Incidence of CMVR-related RD in AIDS Patients.

General RD during the entire course was observed in 24% (95%CI 18%-29%, I^2 =^ 83%) of 2,064 CMVR eyes in 10 studies (see **Figure 4**). The pooled incidence of RD at baseline/enrollment and new RD after anti-CMV therapy was summarized in **Table 4**. For CMVR-related RD, PPV with SO or gas tamponade was the most commonly applied treatment (52%, 95%CI 32%-73%, I^2 =^ 97%), followed by no therapy, laser, and scleral buckle (see **Table 4**). The anatomic success rate of PPV with SO or gas tamponade was 89% (95%CI 85%-93%, I^2 =^ 40%). SO-dependent eyes were observed in 26% (95%CI 0%-92%, I^2 =^ 98%) of patients receiving PPV with SO.

**Table 4:** The pooling results of the incidence, therapy and prognosis of CMVR-related RD in aIDS patients.

Category	No. of studies	Pooled incidence	95% CI		*P*-value	*I* ^2^	Sensitivity analysis	Selected model
Incidence								
*General RD*	*10*	*24%*	*18%*	*29%*	*P<0.01*	*83%*	*Negative*	*Random-effect model*
*RD at baseline/enrollment*	*40*	*13%*	*10%*	*16%*	*P<0.01*	*92%*	*Negative*	*Random-effect model*
*New RD after anti-CMV therapy*	*48*	*12%*	*10%*	*14%*	*P<0.01*	*69%*	*Negative*	*Random-effect model*
Therapy choices for RD								
*PPV with SO or gas tamponade*	*24*	*52%*	*32%*	*73%*	*P<0.01*	*97%*	*Negative*	*Random-effect model*
*Laser*	*3*	*25%*	*4%*	*54%*	*P<0.01*	*94%*	*Negative*	*Random-effect model*
*Scleral buckle*	*2*	*19%*	*9%*	*30%*	*P=0.28*	*14%*	*Negative*	*Fixed-effect model*
*No therapy^a^*	*4*	*46%*	*32%*	*60%*	*P=0.14*	*50%*	*Negative*	*Random-effect model*
Anatomic success rate								
*PPV with SO or gas tamponade*	*12*	*89%*	*85%*	*93%*	*P=0.07*	*40%*	*Negative*	*Fixed-effect model*
*Laser*	*3*	*65%*	*43%*	*84%*	*P=0.13*	*52%*	*Negative*	*Random-effect model*
SO-dependent eyes	3	26%	0%	92%	*P*<0.01	98%	Negative	Random-effect model

a Patients with CMVR-related RD who did not receive any therapy because the retinal reattachment was inoperable or the patients were systemically unwell to undergo surgery.

AIDS, acquired immunodeficiency syndrome; CI, confidence interval; CMV, cytomegalovirus; CMVR, cytomegalovirus retinitis; PPV, pars plana vitrectomy; RD, retinal detachment; SO, silicone oil.

### 3.6 Publication bias

No significant publication bias (P=0.122) was found using Egger’s test.

## 4 Discussion

The present study summarizes information on the demographics, clinical features, treatment, and prognosis of CMVR in AIDS patients. Based on all the currently available evidence, the study found that CMVR is male-dominated, with a high proportion of patients aged <41 years, and is commonly bilateral. CMVR was preponderant in white and non-Hispanic, homosexual AIDS individuals with high HIV RNA load or low CD4+ T-cell count. Blurred vision is the most common symptom, and CMVR may be the initial manifestation leading to AIDS diagnosis. The CMV-DNA positivity rate varied remarkably, being 66%, 87%, and 95% in blood, aqueous humor, and vitreous humor, respectively. The CMVR remission rate can achieve 72%-92% depending on the specific category of anti-CMV therapy. RD is a common complication of CMVR with a general incidence of 24% in the entire course, but PPV with SO or gas tamponade can provide a good anatomical outcome (89%).

### 4.1 Demographics

The findings of our study suggest that CMVR is more common in male or homosexual AIDS patients, which may be explained by the fact that AIDS is more prevalent in men (75%) and men who have sex with men (54%) (Twenty-five years of HIV/AIDS–united states, 1981-2006). The CD4^+^ T-cells of most (78%) CMVR patients in this study count <50 cells/μL. Previous studies have also indicated that CD4^+^ counts <50 cells/μL are significant risk factors for the development of CMVR in AIDS patients (Sittivarakul and Seepongphun, 2018; Munro et al., 2019; Ocieczek et al., 2019). These findings underscore the importance of routine ocular screening for AIDS patients with low CD4^+^ counts. The previously reported incidence of blood CMV load <400 copies/mL varies greatly, from 13% to 91% (Jabs et al., 2004; Shi et al., 2011); the pooled incidence is our study is 62%.

Vitreous CMV-DNA is a highly sensitive marker for CMVR diagnosis, with a pooled positivity of 95% in our study. However, it is only reserved for unusual CMVR cases considering the risk of hemorrhage, infection, and vision loss associated with the sampling procedure. The previously reported aqueous CMV-DNA positivity was inconsistent, ranging from 20% to 100% (Suri et al., 2018; Sittivarakul et al., 2020); the pooled positivity in our study was 87%, suggesting its high sensitivity in diagnosing CMVR. However, its application is still limited since the anterior chamber paracentesis is still invasive and associated with the risks of hemorrhage and infection (Tang et al., 2020).

Therefore, blood CMV-DNA is still important for CMVR diagnosis despite its relatively lower sensitivity (66%). Studies have further explored the cut-off value of blood CMV-DNA for diagnosing CMVR, hopefully enhancing its diagnostic efficacy (Tang et al., 2020). Additionally, it is also effective as a serological indicator for disseminated extraocular CMV diseases (Mizushima et al., 2015). Urine and blood cultures are positive in 67% and 48% of CMVR patients in our study. Previous studies have indicated that urine or blood culture positivity, a possible indicator of higher viral load and more severe immune suppression, is associated with a higher possibility of binocular involvement and a poorer prognosis for CMVR (Jabs et al., 1998b).

### 4.2 Clinical features

In our study, the main symptoms of CMVR included blurred vision, floaters, and visual field defects. We also observed several common symptoms of ocular inflammation in these CMVR patients, including photophobia, watering, and pain, which are easily ignored. Additionally, 29% of the included CMVR patients were asymptomatic, revealing the difficulty in the early diagnosis of CMVR in AIDS patients since patients without ocular symptoms may not seek ophthalmic examinations. A delay in CMVR diagnosis, potentially leading to irreversible vision loss, as well as a delay in identifying and managing life-threatening extraocular CMV infection (such as pneumonitis and encephalitis), is detrimental for CMVR patients. All these findings highlight the importance of routine ophthalmic screening for AIDS patients, even for those with only slight or no ocular discomfort. However, due to the lack of trained ophthalmologists and the enormous burden of AIDS, routine ocular examination is rarely performed in clinical practice, especially in resource-limited developing countries (Ford et al., 2013). A comprehensive guideline for ophthalmic screening in AIDS patients and the training of ophthalmologists are warranted.

The incidence of CMVR as the initial AIDS-defining clinical manifestation remains largely unknown. In this study, CMVR was the clue to AIDS diagnosis in 9% of CMVR patients, indicating a potentially important role of ophthalmologists in identifying HIV infection. Additionally, we found that CMVR was distributed slightly more distributed as “peripheral type” than “central type” (51% versus 49%). Based on this finding, we assumed that an imaging system capturing a wider field might facilitate the identification of peripheral lesions and the diagnosis of CMVR. Recently, Du et al. (2021) applied ultra-wide-field (UWF) retinal imaging for screening AIDS-related CMVR and found excellent concordance, high specificity, and high sensitivity. Further studies applying innovative imaging techniques to evaluate CMVR are needed in the future.

### 4.3 Treatment and prognosis

The availability of cART has revolutionized the picture of CMVR. cART can lower the HIV load and elevate CD4^+^ T-cells, thus restoring immunity to CMV and enabling the discontinuation of anti-CMV therapy without CMVR recurrence (Jabs et al., 1998a; Whitcup et al., 1998). In this study, 85% of the CMVR patients had ever received cART at CMVR diagnosis or at baseline/enrollment. Anti-CMV agents included ganciclovir, valganciclovir, foscarnet, and cidofovir, either administrated systemically or locally. Systemic administration could prevent binocular involvement, control bilateral CMVR, and inhibit extraocular CMV infection development (Arevalo et al., 2005). Local administration could decrease the systemic toxicity of anti-CMV agents and improve patients’ quality of life by obviating the need for an indwelling central catheter (Jabs et al., 2013). In our study, systemic anti-CMV therapy was mostly used (45%), followed by local (35%) and combinational administration (20%).

The pooled CMV remission rate in this study was relatively high, ranging from 72% to 92%, depending on various categories of anti-CMV agents. Although multiple studies have evaluated the efficacy of anti-CMV therapy, difficulty still exists in assessing CMVR prognosis for certain specific anti-CMV agents. In this study, we could not assess the CMVR remission rate of intravenous foscarnet using meta-analysis due to limited studies. Additionally, it seems contradictory that the CMVR remission rate of intravenous combined with intravitreal ganciclovir was lower than single intravenous or intravitreal ganciclovir. This might be partially explained by the limited number of studies included in the meta-analysis.

Discontinuation of maintenance anti-CMV therapy was recommended in CMVR patients with immune recovery, which is regarded as a sustained CD4^+^ T-cells >100 cells/μL for 3-6 months (Kaplan et al., 2009; Holbrook et al., 2011). In this study, 16% of CMVR patients experienced recurrence after discontinuing maintenance anti-CMV therapy. This finding reminds ophthalmologists of the importance of frequent ophthalmological examinations in these patients to detect early reactivation and allow timely resumption of anti-CMV therapy. Additionally, identifying risk factors for CMVR recurrence is also important. Recently, Torriani et al. (2000) have built a model to evaluate the risk of CMVR recurrence, containing the following factors: HIV RNA and CD4^+^ T-cells count at therapy withdrawal, maximal CD4^+^ T-cells count and nadir HIV RNA ever on cART, and the average lymphoproliferative response above 3 SI.

IRU represents the new-onset or increase of intraocular inflammation in the anterior chamber or vitreous of CMVR eyes coincident with immune recovery after initiating cART (Nguyen et al., 2000; Kempen et al., 2006). It is presumed to be mediated by the immune recovery specific to residual CMV antigens in the eye (Otiti-Sengeri et al., 2008). Previous studies have reported IRU as an important cause of visual morbidity in CMVR patients after immune recovery (Thorne et al., 2006b). The pooled incidence of IRU in this study was 26% after initiating cART. Song et al. (2003) found that the risk of IRU was strongly correlated with prior cidofovir use, and thus, they recommended not using cidofovir to reduce the occurrence of IRU. Other factors associated with IRU included the extent of the retinal area involved with CMVR and the presence of vitreous CMV-specific CD8^+^ cells (Karavellas et al., 2001; Mutimer et al., 2002). Further randomized trials are warranted to give more definite information.

### 4.4 CMVR-related RD

CMVR can cause necrosis of the full-thickness retina and retinal pigment epithelium in histology (Friedman, 1984). Then, the vitreous liquefaction and vitreoretinal interface gliosis due to inflammation and traction could easily cause retinal breaks and lead to RD (Brar et al., 2010). In our study, the pooled incidence of CMVR-related general RD in the entire course was 24%, and 12% of CMVR patients experienced new RD after anti-CMV therapy. Ophthalmologists should maintain a focus on patients during their anti-CMV therapy, and regular ocular examinations are helpful. The therapy of CMVR-related RD depends on its location and extent. The present study shows that PPV, laser, and scleral buckle were performed in 52%, 25%, and 19% of CMVR patients, respectively. However, in 4 studies comprising 184 patients, 46% received no therapy because the RD was inoperable or the patients were systemically unwell to undergo surgery (Sandy et al., 1995).

The anatomic success rate of PPV with SO or gas tamponade in CMVR-related RD patients was inconsistent in previous studies, ranging from 57% to 100% (Azen et al., 1998; Martidis et al., 2002; Scott et al., 2003; Almeida et al., 2015). The pooled anatomic reattachment rate in this study was 89%. Previous studies suggested that SO removal is required for optimizing vision and preventing complications of SO in the eyes with SO tamponades (Federman and Schubert, 1988). The pooled incidence of SO-dependent eyes after PPV with SO tamponade in patients with CMVR-related RD was 26%. This finding suggested that, although CMVR-related RD is vision-threatening and troublesome to manage, its prognosis is relatively promising.

### 4.5 Strengths and limitations

To our knowledge, this is the first meta-analysis evaluating all available evidence of CMVR in AIDS patients from different angles. Hopefully, it could provide references for ophthalmologists and facilitate a better understanding of CMVR in AIDS patients.

However, we admitted the existence of several limitations in our study. Firstly, there was substantial heterogeneity across the included studies, which should be considered seriously when interpreting the results of the study. Secondly, the follow-up periods were variable or even not reported in all included studies, which might affect the accuracy and generalizability of the findings. Thirdly, optical coherence tomography (OCT), fundus autofluorescence (FAF), or UWF retinal imaging has recently been applied in the diagnosis and evaluation of CMVR (Yashiro et al., 2018; Sheng et al., 2020; Du et al., 2021). However, signs in these new techniques were not estimated in this study because of the insufficient data in included studies. Finally, the absence of a direct correlation between certain anti-CMV therapy and VA prognosis is also a significant limitation. This may be due to the heterogeneity of the data or the lack of well-designed studies on this topic, although we tried to include all the available data to date. Future research should aim to address these limitations and build on the findings of our study to further reveal their association.

### 4.6 Conclusion

CMVR is a common opportunistic infection with diverse clinical features in AIDS patients. Current therapies for CMVR and CMVR-related RD were shown to be effective. Early detection and routine ophthalmic screening should be promoted in AIDS patients.

## Data availability statement

The original contributions presented in the study are included in the article/**Supplementary Material**. Further inquiries can be directed to the corresponding authors.

## Author contributions

QZ devised this study, carried out the literature search and data extraction, and drafted the manuscript. X-YZ helped in the data extraction, contributed to statistical analysis and revision of the manuscript. N-NL and Y-XC coordinated and participated in the entire process of drafting and revised the manuscript. All authors contributed to the article and approved the submitted version.
