# Supplementary material for: Clinical features of Cytomegalovirus retinitis in patients with acquired immunodeficiency syndrome and efficacy of the current therapy
Source: Front Cell Infect Microbiol. 2023 May 26;13:1107237. doi: 10.3389/fcimb.2023.1107237 (PMC10254806; doi:10.3389/fcimb.2023.1107237)
Supplement: Supplementary file 1 [file DataSheet_1.pdf]

Cytomegalovirus Retinitis in Patients with Acquired Immunodeficiency Syndrome

Demographics

<41

Age <41 years: 57%

Male: 88%
White, non-Hispanic: 60%

Bilateral involvement: 57%

Homosexual: 70%

HIV RNA ≥400 copies/mL: 82%

CD4+ T-cells <50 cells/μL: 78%

Urine CMV culture: 67%
Blood CMV culture: 48%

Blood CMV-DNA: 66%
Aqueous CMV-DNA: 87%
Vitreous CMV-DNA: 95%

Extraocular CMV infection: 18%

Clinical features

Symptoms

- Blurred vision: 55%
- Floaters: 20%
- Asymptomatic: 29%
- Visual field defect: 21%
- Photophobia: 20%
- Pain: 2%
- Watering: 13%

As the clue to AIDS: 9%

Retinal area involved

- 0–25%: 55%
- 25–50%: 30%
- 50–100%: 15%

Location

- Central: 49%
- Peripheral: 51%

Treatment and prognosis

cART: 85%

Anti-CMV therapy

- Systemic: 45%
- Local: 35%
- Combinational: 20%

Prognosis

- CMVR remission: 72%-92%
- CMVR progression: 7%-56%
- CMVR recurrence after discontinuing maintenance anti-CMV therapy: 16%

All-cause mortality: 26%

Immune recovery retinitis: 26%

CMVR-related RD

Incidence

- General RD incidence: 24%
- RD at baseline/enrollment: 13%
- New RD after anti-CMV therapy: 12%

Therapy

- PPV with SO or gas tamponade: 52%
- Laser: 25%
- Scleral buckle: 19%
- No therapy: 46%

Anatomic success

- PPV with SO or gas tamponade: 89%
- Laser: 65%
