# Supplementary material for: Clinical features of Cytomegalovirus retinitis in patients with acquired immunodeficiency syndrome and efficacy of the current therapy
Source: Front Cell Infect Microbiol. 2023 May 26;13:1107237. doi: 10.3389/fcimb.2023.1107237 (PMC10254806; doi:10.3389/fcimb.2023.1107237)
Supplement: Supplementary file 2 [file DataSheet_1.docx]

Supplementary Materials

# Supplementary table 1. PubMed search strategy.

| **# No** | **Searches** |
| --- | --- |
| ***Part I: CMVR*** | |
| 1 | "cytomegalovirus retinitis"[MeSH Terms] OR "cytomegalovirus retinitis"[Title/Abstract] OR "CMV retinitis"[Title/Abstract] OR "CMVR"[Title/Abstract] |
| ***Part II: HIV or AIDS*** | |
| 2 | "hiv"[MeSH Terms] OR "human immunodeficiency virus"[Title/Abstract] OR "Acquired Immunodeficiency Syndrome"[MeSH Terms] OR "AIDS"[Title/Abstract] |
| ***Part III: Study filter*** | |
| 3 | "humans"[MeSH Terms] |
| 4 | "english"[Language] |
| 5 | "meta analysis"[Publication Type] OR "review"[Publication Type] OR "systematic review"[Filter] |
| ***Part IV: Part I, II, and III*** | |
| 6 | 1 and 2 and 3 and 4 |
| 7 | 6 not 5 |

# Supplementary table 2. The Main Characteristics of the Included Studies.

| **No.** | **Author** | **Year** | **Study Design** | **Patients** | **Eyes** | **Age (mean ± SD [range])** | **male (%)** | **Baseline BCVA (median (IQR))** | **Final BCVA (mean ± SD [range])** | **Time lag between HIV and CMVR (median (IQR))** | **Follow-up time (mean [range])** |
| --- | --- | --- | --- | --- | --- | --- | --- | --- | --- | --- | --- |
| 1 | Agrawal | 2022 | retrospective single-center cohort study | 144 | 144 | 46.50 ± 9.77 [23, 72] | 134 (93.1%) | 0.30 (0.18, 0.60) | 0.30 (0.10, 0.60) | 5.79 (2.17, 27.27), m | NR |
| 2 | Xie | 2021 | retrospective single-center cohort study | 80 | 96 | [20, 56] | NR | –1.0 | NR | NR | 24 m |
| 3 | Xie | 2021 | retrospective single-center cohort study | 97 | 138 | 33.73 ± 8.79 [18, 61] | NR | 1 | NR | NR | NR |
| 4 | Sadik | 2021 | retrospective single-center cohort study | 11 | 15 | 47.4 ± 14.3 | 10 (91%) | 0.37 ± 0.54 | NR | NR | 19.4 ± 16.1 m |
| 5 | Moharana | 2021 | retrospective single-center cohort study | 11 | 14 | 39.5 ± 15.4 | 8 (72.7%) | 1.89 ± 1.05 | 1.12 ± 0.55 | NR | 18.45 ± 15.65 m |
| 6 | Kong | 2021 | retrospective single-center cohort study | 37 | 39 | 38.5 ± 9.1 [25, 49] | 35 (94.6%) | 1.3 [0.6, 3.0] | 1.0 [0.6, 3.0] | NR | 6 m |
| 7 | Du | 2021 | retrospective single-center cohort study | 17 | 24 | 39.71 ± 9.66 | 15 (88.2%) | NR | NR | 12 (4, 42), m | NR |
| 8 | Du | 2021 | cross-sectional study | 65 | 65 | 36 (32, 46) | 58 (89.2%) | NR | NR | NR | NR |
| 9 | Du | 2021 | prospective observational study | 18 | 27 | NR | NR | NR | NR | NR | NR |
| 10 | Chen | 2021 | retrospective single-center cohort study | 25 | 25 | 36.73 ± 8.79 | 25 (100.0%) | 0.59 ± 0.39 | NR | NR | NR |
| 11 | Tang | 2020 | retrospective single-center cohort study | 93 | 93 | 35 (28, 44.5) | 85 (91.4%) | 3.8 ± 0.9 | 4.3 ± 0.8 | NR | NR |
| 12 | Sittivarakul | 2020 | retrospective charts review | 45 | 52 | 39.32 ± 8.05 | 23 (51.1%) | 1.20 ± 0.69 | 0.99 ± 0.71 | NR | NR |
| 13 | Singh | 2020 | retrospective chart review | 52 | 87 | 34.7 ± 10.8 [9, 55] | 39 (75.0%) | 0.27 ± 0.32 | NR | 6 [0, 120], m | NR |
| 14 | Sheng | 2020 | retrospective single-center cohort study | 33 | 46 | 39.7 ± 13.9 [21, 78] | 26 (78.8%) | 1.04 ± 0.95 | 0.90 ± 0.94 | NR | 9.6 ± 7.8 m |
| 15 | Murray | 2020 | retrospective chart review | 53 | 72 | 37 (34, 42) | 29 (54.7%) | NR | NR | NR | NR |
| 16 | Mao | 2020 | retrospective chart review | 49 | 49 | 37.14 ± 8.94 | 46 (93.9%) | NR | NR | NR | NR |
| 17 | Ho | 2020 | retrospective chart review | 6 | 7 | NR | NR | 0.55 ± 0.50 | 0.51 ± 0.49 | NR | 6 m |
| 18 | Xie | 2019 | retrospective case series | 31 | NR | 39 ± 9.68 | NR | 0.75 ± 0.29 | NR | NR | NR |
| 19 | Xie | 2019 | retrospective case series | 23 | 32 | NR | NR | central fundus lesion: 1.56 [0.20, 2.30] peripheral fundus lesion: 0.52 [0.20, 1.00] | central fundus lesion: 1.00 [0.00, 1.85] peripheral fundus lesion: 0.19 [0.00, 0.50] | NR | NR |
| 20 | Tsen | 2019 | retrospective single-center cohort study | 28 | 33 | NR | NR | NR | NR | NR | NR |
| 21 | Saini | 2019 | prospective observational study | NR | NR | NR | NR | NR | NR | NR | NR |
| 22 | Ocieczek | 2019 | retrospective chart review | 5 | 5 | 37 [30, 49] | 5 (100.0%) | NR | NR | NR | NR |
| 23 | Holland | 2019 | cross-sectional study | 299 | 401 | 39, median | 242 (81.2%) | NR | NR | NR | NR |
| 24 | Heiden | 2019 | retrospective chart review | 36 | 50 | NR | NR | NR | NR | NR | NR |
| 25 | Yashiro | 2018 | retrospective case series | 11 | 13 | 43.2 ± 6.66 [35, 56] | NR | 1.15 ± 0.40 | 0.86 ± 0.44 | NR | 20.5 ± 17.2 [1, 48] m |
| 26 | Suri | 2018 | retrospective chart review | 15 | 27 | 6, median [6m, 12y] | 10 (66.7%) | NR | 1.58 ± 1.32 | NR | median, 30m |
| 27 | Sittivarakul | 2018 | retrospective single-center cohort study | 132 | 208 | 36 (31,42) median (IQR) | 85 (64.4%) | NR | NR | NR | 0.79 y (IQR 0.23-3.79 y) |
| 28 | Biberg-Salum | 2018 | observational case-control study | 49 | NR | 35 [21, 77] | 38 (77.6%) | NR | NR | NR | NR |
| 29 | Tsen | 2017 | retrospective chart review | 5 | 8 | 43.00 ± 13.32 | 5 (100.0%) | 0.18 ± 0.25 | 1.43 ± 1.20 | NR | 16.2 ± 8.67 |
| 30 | Jabs | 2017 | prospective observational cohort study | 50 | 70 | 36 (32, 44) | 38 (73%) | NR | NR | 1.4 (0.4, 6.0), y | NR |
| 31 | Chen | 2017 | retrospective chart review | 41 | NR | 37.63 ± 9.22 [22, 56] | 37 (90.2%) | 0.64 ± 0.35 | NR | NR | NR |
| 32 | Yeo | 2016 | retrospective chart review | NR | 53 | NR | NR | NR | NR | 283.0 (142.0, 539.5), d | NR |
| 33 | Sittivarakul | 2016 | retrospective longitudinal cohort study | 119 | 119 | 36 (31, 41) | 72 (60.5%) | NR | NR | NR | NR |
| 34 | Liu | 2016 | prospective cross-sectional study | 16 | NR | NR | NR | NR | NR | NR | NR |
| 35 | Leenasirimakul | 2016 | prospective cross-sectional study | 16 | NR | mean, 41.1 (95%CI 36.5-45.7) | 9 (56.3%) | NR | NR | NR | NR |
| 36 | Iyer | 2016 | prospective cross-sectional study | 17 | NR | 49.5 [35, 63] | 16 (94.12%) | NR | NR | NR | NR |
| 37 | Yen | 2015 | case-control study | 164 | 239 | Group1: mean, 37 (95%CI 34-41) Group2: mean, 36 (95%CI 31-40) | 70 (42.68%) | Group1: mean, 0.87 (95%CI 0.68-1.06) Group2: mean, 0.72 (95%CI 0.60-0.80) | NR | NR | NR |
| 38 | Nishijima | 2015 | retrospective single-center study | 24 | NR | 42 (34, 53) median (IQR) | 24 (100.0%) | NR | NR | NR | NR |
| 39 | Mizushima | 2015 | single-center cross-sectional study | 23 |  | 42 (33, 53) median (IQR) | 23 (100.0%) | NR | NR | NR | NR |
| 40 | Mathur | 2015 | retrospective interventional cohort study | 35 | 40 | 35.5, mean | 28 (80.0%) | NR | NR | NR | NR |
| 41 | Jabs | 2015 | prospective observational cohort study | 479 | 667 | 40 (35, 46) | 385 (80.4%) | EDTRS letter: 81 (64, 87), median (IQR) | NR | 1.6 (0.3, 4.8), y | NR |
| 42 | Huang | 2015 | retrospective chart review | 67 | 78 | 38 ± 9 [18, 60] | 58 (86.57%) | NR | NR | NR | [5, 44] m |
| 43 | Wong | 2014 | retrospective chart review | 19 | NR | 45.6 ± 9.35 | 16 (84.2%) | NR | NR | NR | NR |
| 44 | Ruiz-Cruz | 2014 | retrospective chart review | 75 | NR | 33 (29, 39) median (IQR) | 69 (92%) | NR | NR | NR | NR |
| 45 | Kozak | 2014 | longitudinal multicenter observational case–control report | 270 | 357 | 41.0 (35, 46) median (IQR) | 225 (83.3%) | NR | NR | NR | NR |
| 46 | Colby | 2014 | prospective cross-sectional study | 14 | 16 | NR | 10 (71.43%) | NR | NR | NR | NR |
| 47 | Singh | 2013 | retrospective chart review | 26 | 28 | 32, mean | 21 (80.77%) | NR | NR | NR | NR |
| 48 | Shah | 2013 | retrospective chart review | 17 | NR | 43.9 ± 6.0 | 15 (88.2%) | NR | NR | NR | NR |
| 49 | Luo | 2013 | cross-sectional study | 70 | 108 | NR | NR | NR | NR | NR | NR |
| 50 | Jabs | 2013 | prospective cohort study | 250 | 338 | NR | NR | NR | NR | NR | mean, 5.0y; median, 4.3 [1.7, 7.5], y |
| 51 | Wang | 2012 | prospective observational study | 83 | 120 | NR | NR | 0.51 ± 0.63 | NR | NR | NR |
| 52 | Teoh | 2012 | retrospective case series | 224 | NR | 43.0 (34.3, 52.0) median (IQR) | 208 (92.9%) | NR | NR | NR | 26.0 (5.0, 60.0), m |
| 53 | Teoh | 2012 | retrospective case series | 24 | 34 | 46.9 (27, 64) | 21 (87.5%) | NR | NR | NR | median, 95 [10, 829], d; mean, 207.9d |
| 54 | Sugar | 2012 | prospective cohort study | 29 | NR | NR | NR | NR | NR | NR | NR |
| 55 | Oktavec | 2012 | retrospective single-center cohort study | 115 | 166 | 38 [21, 56] median [range] | 74 (64.3%) | median, 20/50; range, 20/10 to NLP | NR | 1.1 [0, 16.7] y, median [range] | NR |
| 56 | Kim | 2012 | retrospective chart review | 22 | 31 | NR | 21 (95.5%) | NR | NR | median, 21 m | median, 38 m |
| 57 | Kempen | 2012 | prospective cohort study | 489 | 729 | NR | NR | NR | NR | NR | NR |
| 58 | Thorne | 2011 | prospective multicenter observational study | 476 | 662 | 41 (35, 46) median (IQR); [17, 68] | 382 (80.2%) | NR | NR | NR | median, 4 [0.5, 9] |
| 59 | Shi | 2011 | retrospective single-center study | 23 | 35 | 41.87 ± 14.27 | 19 (82.61%) | 0.98 ± 0.81 | 0.32 ± 0.77 | NR | NR |
| 60 | Sezgin | 2011 | prospective multicenter observational study | 320 | NR | European Americans: 42.2 ± 7.2; 41.0 (37, 47) African Americans: 38.9 ± 8.1; 38.0 (34, 44) | 262 (81.88%) | NR | NR | NR | NR |
| 61 | O'Connell | 2011 | retrospective descriptive study | 6 | 10 | 6.7 [2-40], months | 2 (33.3%) | NR | NR | NR | NR |
| 62 | Lai | 2011 | retrospective descriptive study | 41 | 51 | NR | NR | median, 6/9 | median, 6/15 | NR | NR |
| 63 | Holbrook | 2011 | prospective observational study | 71 | 101 | group 1: 40 (35, 44) group 2: 44 (37, 49) | 54 (74.6%) | group 1: 75 (46, 86) group 2: 82 (57, 90) | NR | NR | group 1: 6.4 (3.3, 8.1) group 2: 4.5 (3.0, 6.4) |
| 64 | Kunavisarut | 2010 | retrospective interventional cohort study | 35 | NR | NR | 20 (57.14%) | NR | NR | NR | NR |
| 65 | Jabs | 2010 | prospective cohort study | 266 | 351 | 39.0 (34.0, 44.0) | 184 (69.2%) | NR | NR | 23.1 (10.1, 44.6) m | 10.5 (5.4, 22.7) m |
| 66 | Jabs | 2010 | prospective multicenter observational study | 503 | 666 | 41 (35, 46) median (IQR); [17, 68] | 406 (80.7%) | NR | NR | NR | NR |
| 67 | Arantes | 2010 | retrospective chart review | 30 | 44 | 34.8 ± 12.6 | 18 (60.0%) | NR | NR | NR | NR |
| 68 | Holland | 2008 | retrospective observational case series | 125 | 157 | 39 ± 7.5 | 107 (85%) | 0.43 ± 0.71 | NR | NR | NR |
| 69 | Gharai | 2008 | retrospective chart review | 29 | NR | NR | NR | NR | NR | NR | NR |
| 70 | Dujić | 2008 | retrospective chart review | 30 | 47 | 38.8 ± 9.7 | 21 (70.0%) | NR | NR | NR | NR |
| 71 | Martin | 2007 | prospective observational study | 257 | 340 | 39.0 (34.0, 44.0) | 178 (69.3%) | NR | NR | 23.3 (10.4, 45.0), m | 11.2 (5.9, 23.4), m |
| 72 | Jabs | 2007 | retrospective longitudinal cohort study | 360 | 515 | NR | NR | NR | NR | NR | NR |
| 73 | Walmsley | 2006 | prospective cohort study | 23 | 37 | 41, mean | 22 (5.65%) | NR | NR | NR | mean, 33m; median, 34m [5, 61] |
| 74 | Thorne | 2006 | prospective multicenter observational study | 379 | 494 | 41, median | 310 (81.8%) | NR | NR | NR | NR |
| 75 | Kempen | 2006 | retrospective multi-center cohort study | 374 | 539 | NR | 299 (79.95%) | NR | NR | NR | NR |
| 76 | Kappel | 2006 | retrospective chart review | 174 | NR | 40 ± 8.5; 39 [14, 71] | 154 (92%) | 20/30 [20/15, NLP] | NR | NR | NR |
| 77 | Wohl | 2005 | nonrandomized prospective study | 38 | 53 | 40 [22, 55] | 35 (92%) | NR | NR | NR | median, 64 [0, 104], w |
| 78 | Ortega-Larrocea | 2005 | retrospective charts review | 43 | 57 | group 1: mean, 35.4; group 2: mean, 35.1 | 40 (93.0%) | NR | NR | NR | NR |
| 79 | Morrison | 2005 | retrospective interventional case series | 14 | 15 | 44 ± 7.8 | 14 (100.0%) | NR | NR | NR | NR |
| 80 | Kempen | 2005 | single-center cohort study | 589 | 802 | NR | 475 (80.6%) | NR | NR | NR | median, 6.6 [0.07, 59.6], m |
| 81 | Kahraman | 2005 | retrospective charts review | 58 | NR | NR | NR | NR | NR | NR | NR |
| 82 | Jabs | 2005 | prospective multicenter observational study | 165 | NR | 38 (34, 43) median (IQR) | 109 (66.1%) | NR | NR | NR | median, 10.6 (3.7, 21.4), m |
| 83 | Arevalo | 2005 | prospective interventional case series | 13 | 18 | mean, 39 [27, 64] | 11 (84.6%) | NR | NR | median, 27.7 [2, 90], m | mean, 15.6 m |
| 84 | Yust | 2004 | multicentre prospective cohort study | 216 | NR | NR | 180 (83.3%) | NR | NR | NR | NR |
| 85 | Jabs | 2004 | prospective multicenter observational study | 271 | 368 | mean, 41 | 224 (82.8%) | NR | NR | NR | NR |
| 86 | Hodge | 2004 | case-control study | 120 | NR | mean, 38.6 | 109 (90.8%) | NR | NR | NR | NR |
| 87 | Dunn | 2004 | prospective observational study | 42 | 56 | mean, 39.4 | 33 (79%) | NR | NR | median, 34 m | NR |
| 88 | Borucki | 2004 | randomized controlled trials | 82 | NR | median, 38 | 67 (82%) | NR | NR | NR | NR |
| 89 | Yamamoto | 2003 | / | 42 | 35 | NR | NR | NR | NR | NR | NR |
| 90 | Song | 2003 | observational cohort study | 43 | 64 | NR | 38 (88.4%) | NR | NR | NR | NR |
| 91 | Shane | 2003 | retrospective chart review | 24 | 40 | mean, 40 [29, 51] | 20 (83.3%) | median, 20/25 [20/16, LP] | 20/80 [20/20, FC] | NR | NR |
| 92 | Kempen | 2003 | multicentre prospective cohort study | 262 | NR | group 1: 41.7; group 2: 37.2 | 213 (81.3%) | NR | NR | NR | NR |
| 93 | Kempen | 2003 | prospective observational study | 589 | 669 | NR | 477 (81%) | NR | NR | NR | NR |
| 94 | Jabs | 2003 | prospective cohort study | 197 | 272 | mean, 38.3 [22, 68] | 142 (72.1%) | NR | NR | median, 22.5 m | median, 9.2 [0.7, 76.7], m |
| 95 | Goldberg | 2003 | prospective observational study | 63 | 90 | 32.3 ± 1.2 | 58 (92%) | NR | NR | NR | NR |
| 96 | Yamamoto | 2002 | / | 37 | NR | NR | NR | NR | NR | NR | NR |
| 97 | Wei | 2002 | retrospective chart review | 138 | 183 | 36 [2, 55] | 121 (88.0%) | NR | NR | NR | NR |
| 98 | Martin | 2002 | randomized controlled trials | 160 | 200 | group1: 36.5 ± 7.3; group2: 39.0 ± 7.6 | 145 (90.6%) | NR | NR | NR | NR |
| 99 | Lalezari | 2002 | randomized controlled trials | 281 | 408 | group1: 39 ± 8; group2: 39 ± 8; group3: 39 ± 7; group4: 39 ± 6 | 248 (88.3%) | NR | NR | NR | NR |
| 100 | Jabs | 2002 | randomized controlled trials | 83 | 130 | mean, 39.6 | 76 (91.6%) | NR | NR | NR | NR |
| 101 | Berenguer | 2002 | prospective observational study | 35 | 44 | 38 (34, 39), median (IQR) | 27 (77.1%) | NR | NR | NR | 90 (53, 97), w, median(IQR) |
| 102 | THE VITRAVENE STUDY GROUP | 2002 | randomized controlled trials | 93 | 106 | group 1: median, 35; group 2: median, 41; group 3: median, 35; group 4: median, 35 | 83 (89.2%) | NR | NR | NR | NR |
| 103 | THE VITRAVENE STUDY GROUP | 2002 | randomized controlled trials | 29 | NR | median, 36 | 27 (93.1%) | NR | NR | NR | NR |
| 104 | Zambarakji | 2001 | longitudinal cohort study | 53 | NR | NR | NR | NR | NR | NR | NR |
| 105 | Reed | 2001 | retrospective case series | 8 | NR | NR | NR | NR | NR | NR | mean, 15.5 [11, 20], m |
| 106 | López-Cortés | 2001 | / | 27 | 34 | mean, 33; [25, 47] | 18 (66.7%) | NR | NR | NR | 33 ± 9, w |
| 107 | Kempen | 2001 | prospective observational study | 511 | 773 | group 1: median, 38; SD, 8.4; group 2: median, 37; SD, 7.6; group 3: median, 38; SD, 8.2 | 423 (82.8%) | NR | NR | NR | NR |
| 108 | Torriani | 2000 | prospective observational study | 17 | NR | median, 20 [27, 63] | 15 (88%) | NR | NR | NR | NR |
| 109 | Robinson | 2000 | prospective observational study | 16 | 23 | 39.9 ± 5.9 | NR | NR | NR | NR | NR |
| 110 | Raina | 2000 | retrospective case series | 26 | 41 | mean, 38 [32, 58] | 25 (96.2%) | NR | NR | mean, 54.4 [1, 144], m | NR |
| 111 | Pauriah | 2000 | retrospective case series | 27 | NR | 40.8 ± 6.3 | 25 (92.6%) | NR | NR | median, 1.5 y | NR |
| 112 | Muccioli | 2000 | prospective observational study | 26 | 39 | 35.2 [26, 47] | 23 (88.5%) | NR | NR | NR | mean, 3.7 m [30 d, 9 m] |
| 113 | Macdonald | 2000 | consecutive noncomparative case series | 22 | NR | 40 ± 9 | 17 (77.3%) | NR | NR | NR | NR |
| 114 | Jalali | 2000 | retrospective chart review | 44 | NR | NR | NR | NR | NR | NR | NR |
| 115 | Jacobson | 2000 | retrospective case series | 14 | NR | 37.8 ± 5.4 | 10 (71.4%) | NR | NR | NR | NR |
| 116 | Holbrook | 2000 | randomized controlled trials | 224 | 318 | NR | NR | NR | NR | NR | NR |
| 117 | Berenguer | 2000 | retrospective longitudinal cohort study | 51 | 82 | median, 34 | 10 (19.6%) | NR | NR | NR | NR |
| 118 | The Studies of Ocular Complications of AIDS Research Group in collaboration with the AIDS Clinical Trials Group | 2000 | randomized controlled trials | 58 | 71 | mean, 39.1 | 53 (91.4%) | NR | NR | NR | median, 0.89 person-year |
| 119 | Wutoh | 1999 | retrospective chart review | 212 | NR | median, 35.5 | 176 (83.0%) | NR | NR | NR | NR |
| 120 | Whitcup | 1999 | prospective nonrandomized interventional trial | 14 | 20 | 40.1 ± 6.3 | NR | NR | NR | NR | 16.5 ± 4.2, m |
| 121 | Smith | 1999 | experiment | 27 | NR | median, 40 [26, 62] | 25 (92.6%) | NR | NR | NR | NR |
| 122 | Roth | 1999 | retrospective chart review | 54 | 88 | 39.0 ± 7.4 | 42 (77.8%) | median, 20/32 | NR | 9.3 ± 8.7, m | NR |
| 123 | Postelmans | 1999 | prospective observational study | 8 | 13 | mean, 42 [31, 54] | 8 (100.0%) | NR | NR | median, 613 [326, 787], d | median, 248 [183, 368], d |
| 124 | Plummer | 1999 | retrospective observational case series | 17 | 21 | NR | NR | NR | NR | NR | NR |
| 125 | Martin | 1999 | randomized controlled trials | 377 | 459 | group 1: median, 38; group 2: median, 38; group 3: median, 37; | 355 (94.2%) | NR | NR | NR | NR |
| 126 | Lim | 1999 | retrospective chart review | 63 | 82 | median, 39 [28, 61] | 62 (98.4%) | median, 20/25 [20/20, 20/200] | NR | NR | median, 6 m; [2 w, 3 y] |
| 127 | Lee | 1999 | retrospective chart review | 24 | 38 | median, 36.5 [29, 45] | 23 (95.8%) | median, 6/9 [6/5, HM] | NR | NR | median, 323 [30, 901], d |
| 128 | Karavellas | 1999 | retrospective chart review | 30 | NR | NR | NR | NR | NR | NR | median, 21.5 [6, 46], m |
| 129 | Jacobson | 1999 | phase 1 trial | 7 | NR | 38.6 ± 4.2 | 6 (85.7%) | NR | NR | NR | NR |
| 130 | Hutchinson | 1999 | retrospective chart review | 13 | 21 | NR | NR | NR | NR | NR | NR |
| 131 | Henderson | 1999 | retrospective chart review | 80 | NR | NR | NR | NR | NR | NR | NR |
| 132 | Guembel | 1999 | uncontrolled case series | 49 | 79 | 34 ± 7.6 [13, 63] | 41 (83.7%) | NR | NR | NR | 25 ± 10 [2, 34], mo |
| 133 | Doan | 1999 | single-center cross-sectional study | 131 | NR | NR | NR | NR | NR | NR | NR |
| 134 | Davis | 1999 | retrospective cohort study | 53 | NR | group1: 38.6 ± 7.6 [31, 53] group2: 38.6 ± 7.6 [10, 53] | NR | NR | NR | NR | NR |
| 135 | Cochereau | 1999 | retrospective case series | 10 | 16 | 41 ± 6 [ 35, 52] | 10 (100.0%) | NR | NR | NR | median, 8 [1, 12], m |
| 136 | Cassoux | 1999 | retrospective case series | 9 | 14 | mean, 39 [29, 53] | 8 (88.9%) | NR | NR | NR | NR |
| 137 | Bainbridge | 1999 | retrospective chart review | 26 | NR | mean, 38.4 [31, 58] | 25 (96.2%) | NR | NR | NR | NR |
| 138 | Ambati | 1999 | retrospective chart review | 17 | 23 | group 1: 38.1 ± 5.5; group 2: 40.6 ± 4.4 | 14 (82.4%) | group 1: 0.121 ± 0.178; group 2: 0.164 ± 0.148 | NR | NR | NR |
| 139 | Zegans | 1998 | retrospective chart review | 8 | NR | 37.5 ± 5.6 | 7 (87.5%) | NR | NR | NR | NR |
| 140 | Wutoh | 1998 | retrospective chart review | 194 | NR | mean, 36 | 154 (80%) | NR | NR | NR | NR |
| 141 | Walsh | 1998 | longitudinal cohort study | 147 | NR | 38.4 ± 7.9 | 140 (95.2%) | NR | NR | NR | NR |
| 142 | Verbraak | 1998 | experiment | 58 | NR | mean, 40 [27, 49] | NR | NR | NR | mean, 2.5 [1, 6], y | NR |
| 143 | Tural | 1998 | retrospective cohort study | 7 | NR | median, 33 [30, 43] | 6 (85.7%) | NR | NR | median, 28 m | median, 9 [9, 12], m |
| 144 | Macdonald | 1998 | / | 11 | 17 | 42 ± 3.1 | 10 (91%) | NR | NR | NR | NR |
| 145 | Jabs | 1998 | prospective observational study | 122 | NR | median, 39 | 94 (77%) | NR | NR | NR | NR |
| 146 | Jabs | 1998 | prospective observational study | 108 | NR | median, 39 | 84 (77.8%) | NR | NR | NR | NR |
| 147 | Dhillon | 1998 | retrospective chart review | 9 | 16 | NR | 7 (77.8%) | NR | NR | NR | NR |
| 148 | Casado | 1998 | retrospective cohort study | 17 | NR | mean, 37 [30, 55] | 10 (58.8%) | NR | NR | median, 18 [1, 96], m | median, 17 m |
| 149 | Akler | 1998 | retrospective cohort study | 18 | 30 | mean, 38.5 [30, 52] | 17 (94.4%) | NR | NR | NR | NR |
| 150 | Vrabec | 1997 | retrospective cohort study | 14 | NR | NR | NR | group 1: mean, 20/20; group 2: mean, 20/25 | NR | NR | group 1: mean, 9 [2, 19], m; group 2: mean, 10.3 [2, 24] |
| 151 | Tay-Kearney | 1997 | case-control study | 49 | NR | 40.3 ± 8.4 | 42 (86%) | NR | NR | NR | NR |
| 152 | Taskintuna | 1997 | nonrandomized consecutive case series | 35 | 51 | group 1: 38.7 [31, 52]; group 2: 40.3 [34, 56] | 32 (91.4%) | NR | NR | NR | group 1: mean, 65.3 [17, 140]; group 2: mean, 87.8 [38, 186] |
| 153 | Rasmussen | 1997 | prospective observational study | 19 | NR | 43 ± 6 | 18 (94.7%) | NR | NR | NR | NR |
| 154 | Musch | 1997 | randomized controlled trials | 173 | 236 | group 1: 39.4 ± 7.2; group 2: 38.1 ± 7.3; group 3: 39.2 ± 5.7 | 160 (92.5%) | NR | NR | NR | group 1 & 2: median, 156 d; group 3: median, 81 d |
| 155 | Magone | 1997 | case-control study | 31 | NR | 39.7 ± 1.0 | 29 (93.5%) | NR | NR | NR | NR |
| 156 | Latkany | 1997 | case-control study | 6 | NR | 40.1 ± 9.0 | 6 (100%) | 0.11 ± 0.07 | NR | NR | NR |
| 157 | Lalezari | 1997 | randomized controlled trials | 48 | 59 | group 1: median, 38; group 2: median, 38 | 46 (95.8%) | NR | NR | NR | NR |
| 158 | Jacobson | 1997 | retrospective case series | 5 | 6 | 40.8 ± 5.9 | 5 (100%) | NR | NR | NR | 7.8 ± 2.8, m |
| 159 | Davis | 1997 | nonrandomized observational cohort study | 63 | 109 | median, 38 [25, 60] | 57 (90%) | 0.53 ± 0.78 | 0.99 ± 1.04 | NR | NR |
| 160 | Danise | 1997 | experiment | 5 | NR | 34.4 ± 5.3 | 5 (100%) | NR | NR | NR | NR |
| 161 | Burke | 1997 | multicenter retrospective cohort study | 805 | NR | NR | 749 (93.0%) | NR | NR | NR | NR |
| 162 | Holbrook | 1997 | multicenter randomized trials | 195 | 389 | group 1: median, 39, IRQ, (36, 44); group 2: median, 38, IQR (35, 43) | 175 (89.7%) | ETDRS letters: group 1: median, 85, IQR, (80, 89); group 2: median, 85, IQR (75, 90) | NR | NR | NR |
| 163 | Studies of Ocular Complications of AIDS (SOCA) in collaboration with the AIDS Clinical Trial Group | 1997 | multicenter randomized trials | 207 | NR | NR | NR | NR | NR | NR | NR |
| 164 | Wu | 1996 | observational cross-sectional study | 26 | 41 | 38.5 ± 5.9 | 25 (96%) | NR | NR | 29.14 ± 17.5 m | NR |
| 165 | Rahhal | 1996 | prospective nonrandomized consecutive case series | 22 | 32 | mean, 37.7 [27, 57] | 22 (95%) | ETDRS letters: mean, 20/24 | NR | NR | NR |
| 166 | Pannuti | 1996 | retrospective cohort study | 24 | 33 | median, 35.0 | 20 (83.3%) | NR | NR | NR | NR |
| 167 | Mallolas | 1996 | prospective clinical study | 33 | NR | NR | NR | NR | NR | NR | NR |
| 168 | Hoover | 1996 | cohort study | 73 | NR | NR | NR | NR | NR | NR | NR |
| 169 | Hodge | 1996 | single-arm descriptive study | 40 | 57 | median, 37.5 | 40 (100%) | NR | NR | NR | median, 53.1 [6,156] w |
| 170 | Flores-Aguilar | 1996 | prospective clinical study | 52 | NR | NR | NR | NR | NR | NR | NR |
| 171 | Bowen | 1996 | prospective clinical study | 45 | NR | median, 34 [26, 62] | 37 (82.2%) | NR | NR | NR | NR |
| 172 | Baudouin | 1996 | consecutive noncomparative case series | 111 | 156 | mean, 32 [14, 53] | 77 (69.4%) | NR | NR | NR | 19.8 ± 2.9, w [1 w, 32 m] |
| 173 | The Cytomegalovirus Retreatment Trial | 1996 | multicenter randomized controlled trials | 279 | 453 | mean, 38.6 | 256 (91.8%) | mean, 76 ETDRS letters | NR | NR | NR |
| 174 | Spaide | 1995 | cross-sectional study | 67 | NR | NR | NR | NR | NR | NR | NR |
| 175 | Sandy | 1995 | cross-sectional study | 147 | NR | NR | NR | NR | NR | NR | NR |
| 176 | McCann | 1995 | experiment | 59 | NR | NR | NR | NR | NR | NR | NR |
| 177 | Lowder | 1995 | case review study | 17 | NR | 35.1 ± 6.4 | 17 (100%) | NR | NR | NR | NR |
| 178 | Kirsch | 1995 | consecutive case series | 17 | 24 | mean, 40.3 [34, 56] | 16 (94.4%) | NR | NR | NR | mean, 87.8 [38, 186], d |
| 179 | Jabs | 1995 | retrospective chart review | 287 | 435 | mean, 38 [16, 67] | 253 (88%) | NR | NR | 14 ± 11 [0, 81], m | NR |
| 180 | Holland | 1995 | retrospective chart review | 18 | 24 | group 1: 45.0 ± 9.6; group 2: 36.1 ± 6.8 | 18 (100%) | NR | NR | NR | group 1: 71.5 ± 36.5, d; group 2: 134.3 ± 60.7, d |
| 181 | Drew | 1995 | open-label randomized study | 117 | 158 | group 1: mean, 37.7; group 2: mean, 40.4 | 112 (95.7%) | NR | NR | NR | NR |
| 182 | Bylsma | 1995 | consecutive case series | 24 | 38 | NR | NR | NR | NR | NR | NR |
| 183 | Brody | 1995 | consecutive case series | 21 | 32 | [27, 52] | 20 (95.2%) | NR | NR | NR | NR |
| 184 | Bloom | 1995 | prospective cohort study | 147 | 197 | 39 ± 7 [24, 61] | 143 (7.3%) | median, 6/9 [6/5, NPL] | median, 6/18 [6/5, NPL] | NR | mean, 30 w [6 w, 5 y] |
| 185 | Studies of Ocular Complications of AIDS Research Group in collaboration with AIDS Clinical Trials Group | 1995 | randomized controlled trials | 150 | 211 | group 1: median,39, SD, 8; group 2: median,37, SD, 6 | 134 (89.3%) | NR | NR | group 1: median 11, SD, 15; group 2: m,17, SD, 17, m | NR |
| 186 | The Oral Ganciclovir European and Australian Cooperative Study Group | 1995 | randomized controlled trials | 159 | 205 | group 1: mean, 38.6 [23, 58]; group 2: mean, 39.4 [25, 62] | 149 (93.7%) | NR | NR | NR | NR |
| 187 | Martin | 1994 | randomized controlled trials | 26 | 30 | group 1: 40.6 ± 3.6; group 2: 38.9 ± 8.7; group 3: 41.5 ± 5.4 | 25 (96.2%) | NR | NR | NR | median survival, 295 d |
| 188 | Kuppermann | 1994 | prospective clinical study | 51 | 65 | mean, 38 [28, 62] | NR | mean, 20/60 [20/20, LP] | mean, 20/100 [20/25, NLP] | NR | mean, 20 [1, 138], w |
| 189 | Jacobson | 1994 | multicenter randomized trials | 156 | 262 | group 1: median, 26; group 2: median, 35; group 3: median, 37 | 145 (94%) | NR | NR | NR | NR |
| 190 | Jacobson | 1994 | randomized controlled trials | 29 | NR | mean, 40 | 27 (93%) | NR | NR | NR | NR |
| 191 | Hardy | 1994 | randomized controlled trials | 53 | NR | group 1: mean, 38; group 2: mean, 39 | 52 (98.1%) | NR | NR | NR | NR |
| 192 | Gerna | 1994 | retrospective case series | 65 | NR | NR | NR | NR | NR | NR | NR |
| 193 | Diaz-Llopis | 1994 | retrospective case series | 11 | 15 | mean, 36.6 [29, 44] | 9 (81.8%) | 0.42 ± 0.30 | 0.83 ± 0.94 | mean, 11.2 [4, 24], m | 15.8 ± 6.97, w |
| 194 | Studies of Ocular Complications of AIDS Research Group in collaboration with AIDS Clinical Trials Group | 1994 | randomized controlled trials | 224 | 327 | median, 37, IQR, (32, 43) | 204 (91.1%) | median, 84, IQR, (78, 88) | NR | NR | NR |
| 195 | Spector | 1993 | multicenter randomized controlled trials | 35 | 49 | group 1: mean, 37.0 [25, 47]; group 2: mean, 37.5 [21, 50] | 33 (94.3%) | NR | NR | NR | 19.3 ± 4.1, d |
| 196 | Roarty | 1993 | retrospective case series | 22 | 39 | [24, 54] | 22 (100%) | NR | NR | NR | NR |
| 197 | Kuppermann | 1993 | retrospective case series | 22 | 23 | median, 41 [31, 62] | 18 (81.8%) | NR | NR | NR | NR |
| 198 | Kuppermann | 1993 | prospective clinical study | 26 | NR | NR | NR | NR | NR | NR | NR |
| 199 | Jacobson | 1993 | clinical trial | 48 | NR | NR | 48 (100%) | NR | NR | NR | NR |
| 200 | Flores-Aguilar | 1993 | case-control study | 11 | 21 | median, 36.3 [31, 41] | 9 (81.8%) | NR | NR | NR | NR |
| 201 | Dhillon | 1993 | retrospective chart review | 24 | 32 | group 1: mean, 30.9; group 2: mean, 36.2 | 19 (79.2%) | NR | NR | NR | [2, 26], m |
| 202 | Young | 1992 | retrospective chart review | 23 | 37 | NR | NR | NR | NR | mean, 12.6 [1, 31], m | NR |
| 203 | Regillo | 1992 | retrospective case series | 11 | NR | 37.0 ± 9.2 | 11 (100%) | 1.54 ± 1.13 | 2.07 ± 0.78 | NR | 15.4 ± 9.7, w |
| 204 | Pertel | 1992 | retrospective chart review | 52 | 84 | 34.7 ± 10.8 [9, 55] | 39 (75%) | 0.27 ± 0.32 | NR | median, 6 [0, 120], m | NR |
| 205 | Moyle | 1992 | randomized controlled trials | 62 | NR | mean, 37.4 | NR | NR | NR | NR | mean, 31.5, w |
| 206 | Freeman | 1992 | consecutive case series | 24 | 29 | mean, 36.8 [29, 62] | NR | mean, 20/125 [20/25, LP] | mean, 20/70 [20/25, NLP] | NR | mean, 29 [13, 125], w |
| 207 | Cheong | 1992 | retrospective chart review | 17 | 17 | group 1: mean, 28 years; group 2: mean, 36 years | 12 (70.6%) | NR | NR | NR | NR |
| 208 | Bernauer | 1992 | retrospective chart review | 8 | 8 | mean, 41 [29, 53] | 8 (100.0%) | NR | NR | NR | NR |
| 209 | Studies of Ocular Complications of AIDS Research Group in collaboration with AIDS Clinical Trials Group | 1992 | randomized controlled trials | 234 | 234 | NR | NR | NR | NR | NR | NR |
| 210 | Sha | 1991 | single-arm phase II pilot trials | 10 | NR | mean, 38 | 10 (100.0%) | NR | NR | NR | NR |
| 211 | Peters | 1991 | retrospective chart review | 58 | NR | NR | NR | NR | NR | NR | NR |
| 212 | Palestine | 1991 | randomized controlled trials | 24 | NR | group 1: 34.8 ± 4.43; group 2: 42.3 ± 7.17 | NR | mean, 20/25 | NR | NR | NR |
| 213 | Orellana | 1991 | retrospective chart review | 31 | 39 | NR | NR | [20/20, LP] | [20/20, NLP] | NR | NR |
| 214 | Hall | 1991 | retrospective case series | 63 | NR | mean,39.8 [23, 68] | 63 (100%) | NR | NR | 10.0 ± 8.2, m | 7.4 ± 4.2, m |
| 215 | Dugel | 1991 | retrospective case series | 19 | 22 | 38.3 ± 6.3 | NR | NR | NR | NR | NR |
| 216 | Cochereau-Massin | 1991 | prospective open study | 44 | 64 | mean, 38.4 [28, 62] | 41 (93.2%) | NR | NR | mean, 19 [3, 36], m | NR |
| 217 | Millar | 1990 | retrospective chart review | 16 | NR | mean, 36.4 [27, 47] | 16 (100.0%) | NR | NR | NR | NR |
| 218 | Jennens | 1990 | retrospective chart review | 14 | NR | NR | NR | NR | NR | NR | NR |
| 219 | Jacobson | 1990 | retrospective case series | 6 | NR | NR | 6 (100%) | NR | NR | NR | NR |
| 220 | Gross | 1990 | prospective clinical study | 67 | 95 | NR | 66 (98.5%) | NR | NR | mean, 15 m | mean, 15.6 w |
| 221 | Lehoang | 1989 | retrospective case series | 31 | 41 | 35.4 ± 6.5 | 28 (90.3%) | NR | NR | 7.7 ± 5.8, m | NR |
| 222 | Jacobson | 1989 | retrospective case series | 10 | NR | median, 36 [25, 43] | NR | NR | NR | NR | NR |
| 223 | Jabs | 1989 | retrospective chart review | 46 | 62 | mean, 39 [24, 60] | 42 (91%) | NR | NR | group 1: mean, 11 m; group 2: mean, 8 m | NR |
| 224 | Heinemann | 1989 | retrospective chart review | 7 | 13 | 39.3 ± 3.9 [33, 45] | 6 (85.7%) | NR | NR | mean, 12.5 m | [14, 56], w |
| 225 | Cantrill | 1989 | retrospective chart review | 10 | 17 | [29, 49] | 10 (100%) | 0.41 ± 0.74 | 0.27 ± 0.32 | 0.64 ± 0.86 | 124.8 ± 48.6, d |
| 226 | Walmsley | 1988 | retrospective chart review | 13 | NR | [25, 48] | 13 (100.0%) | NR | NR | NR | NR |
| 227 | Ussery | 1988 | retrospective chart review | 11 | 14 | [26, 42] | 11 (100%) | [20/20, HM] | NR | NR | NR |
| 228 | Orellana | 1988 | retrospective case series | 6 | 11 | [29, 55] | 5 (83.3%) | [20/30, NLP] | [20/30, NLP] | NR | NR |
| 229 | Jacobson | 1988 | randomized prospective comparative study | 25 | NR | 41 ± 10 | 25 (100%) | NR | NR | NR | 5.0 ± 2.6, m |
| 230 | Buhles | 1988 | retrospective cohort study | 164 | NR | NR | NR | NR | NR | NR | NR |
| 231 | Orellana | 1987 | retrospective chart review | 41 | 66 | [27, 55] | 38 (92.7%) | NR | NR | mean, 7.8 m [3 w, 20 m | NR |
| 232 | Laskin | 1987 | retrospective chart review | 67 | 127 | NR | NR | NR | NR | NR | NR |
| 233 | Jabs | 1987 | retrospective chart review | 17 | 24 | 38.2 ± 8.2 | 16 (94.1%) | [20/20, NLP] | [20/20, NLP] | NR | NR |
| 234 | Henderly | 1987 | prospective clinical study | 23 | 35 | 37.3 ± 7.8 | NR | NR | NR | NR | NR |
| 235 | Collaborative DHPG treatment study group | 1986 | retrospective cohort study | 14 | NR | 36.8 ± 4.9 | 14 (100%) | NR | NR | NR | NR |
| 236 | Pepose | 1985 | consecutive case series | 12 | 18 | 34.4 ± 4.9 | 12 (100%) | NR | NR | NR | NR |

**Abbreviations:** BCVA: best corrected visual acuity; d: days; ETDRS: Early Treatment Diabetic Retinopathy Study; FC: figure count; HM: hand movement; IQR: interquartile range; LP: light perception; m: months; NLP: no light perception; NR: not reported; SD: standard deviation; w: weeks; y: years.

# Supplementary table 3. The Pooling Results of the Therapy of CMVR in AIDS Patients in the Induction Stage and Maintenance Stage.

| **Category** | **No. of studies** | **Pooled incidence** | **95% CI** | | ***P*-value** | ***I*^2^** | **Sensitivity analysis** | **Selected model** |
| --- | --- | --- | --- | --- | --- | --- | --- | --- |
| **Anti-CMV therapy** |  |  |  |  |  |  |  |  |
| ***Induction stage*** |  |  |  |  |  |  |  |  |
| *Intravenous and intravitreal ganciclovir* | *5* | *39%* | *3%* | *84%* | *P<0.01* | *98%* | *Negative* | *Random-effect model* |
| *Intravitreal ganciclovir* | *12* | *76%* | *52%* | *93%* | *P<0.01* | *97%* | *Negative* | *Random-effect model* |
| *Intravenous ganciclovir* | *23* | *75%* | *62%* | *86%* | *P<0.01* | *97%* | *Negative* | *Random-effect model* |
| *Intravenous foscarnet* | *13* | *57%* | *36%* | *77%* | *P<0.01* | *98%* | *Negative* | *Random-effect model* |
| *Intravenous cidofovir* | *4* | *86%* | *15%* | *100%* | *P<0.01* | *99%* | *Negative* | *Random-effect model* |
| ***Maintenance stage*** |  |  |  |  |  |  |  |  |
| *Intravitreal ganciclovir* | *7* | *80%* | *42%* | *100%* | *P<0.01* | *98%* | *Negative* | *Random-effect model* |
| *Intravenous ganciclovir* | *22* | *61%* | *49%* | *73%* | *P<0.01* | *97%* | *Negative* | *Random-effect model* |
| *Intravenous ganciclovir and foscarnet* | *3* | *50%* | *3%* | *97%* | *P<0.01* | *98%* | *Negative* | *Random-effect model* |
| *Oral ganciclovir* | *9* | *62%* | *32%* | *88%* | *P<0.01* | *98%* | *Negative* | *Random-effect model* |
| *Oral valganciclovir* | *3* | *40%* | *5%* | *83%* | *P<0.01* | *95%* | *Negative* | *Random-effect model* |
| *Intravenous foscarnet* | *13* | *65%* | *44%* | *83%* | *P<0.01* | *98%* | *Negative* | *Random-effect model* |
| *Intravenous cidofovir* | *4* | *86%* | *15%* | *100%* | *P<0.01* | *99%* | *Negative* | *Random-effect model* |

**Abbreviations:** AIDS: acquired immunodeficiency syndrome; CI: confidence interval; CMV: cytomegalovirus; CMVR: cytomegalovirus retinitis.

# Supplementary table 4. The Pooling Results of the Postoperative Complications in CMVR Patients after Receiving Intraocular Ganciclovir Implant.

| **Category** | **No. of studies** | **Pooled incidence** | **95% CI** | | ***P*-value** | ***I*^2^** | **Sensitivity analysis** | **Selected model** |
| --- | --- | --- | --- | --- | --- | --- | --- | --- |
| **Cataract** | *7* | *10%* | *4%* | *19%* | *P<0.01* | *86%* | *Negative* | *Random-effect model* |
| **Endophthalmitis** | *7* | *0%* | *0%* | *0%* | *P=0.09* | *45%* | *Negative* | *Fixed-effect model* |
| **Epiretinal membrane** | *4* | *4%* | *2%* | *6%* | *P=0.14* | *45%* | *Negative* | *Fixed-effect model* |
| **Hypotony** | *4* | *4%* | *0%* | *10%* | *P<0.01* | *79%* | *Negative* | *Random-effect model* |
| **Macular edema** | *5* | *4%* | *1%* | *7%* | *P=0.03* | *62%* | *Negative* | *Random-effect model* |
| **Retinal tear** | *3* | *6%* | *3%* | *10%* | *P=0.49* | *0%* | *Negative* | *Fixed-effect model* |
| **Uveitis** | *4* | *4%* | *2%* | *6%* | *P=0.85* | *0%* | *Negative* | *Fixed-effect model* |
| **Vitreous hemorrhage** | *7* | *8%* | *6%* | *10%* | *P=0.07* | *49%* | *Negative* | *Fixed-effect model* |

Abbreviations: CI: confidence interval; CMVR: cytomegalovirus retinitis.
